# Stratigraphic reconstruction of the Víti breccia at Krafla volcano (Iceland): insights into pre-eruptive conditions priming explosive eruptions in geothermal areas

**DOI:** 10.1007/s00445-021-01502-y

**Published:** 2021-11-02

**Authors:** Cristian Montanaro, Anette Kærgaard Mortensen, Tobias B. Weisenberger, Donald B. Dingwell, Bettina Scheu

**Affiliations:** 1 Earth and Environmental Sciences, Ludwig-Maximilians-Universität München, Theresienstrasse 41, 80333 Munich, Germany; 2 Háaleitisbraut 68, 110 Reykjavík, Iceland; 3 Research Centre Breiðdalsvík, University of Iceland, Gamla Kaupfélagið, 760 Breiðdalsvík, Iceland

**Keywords:** Magmatic-hydrothermal eruption, Geothermal, Krafla volcano, Víti, Rhyolite, Hydrothermal alteration, Eruption mechanisms

## Abstract

Krafla central volcano in Iceland has experienced numerous basaltic fissure eruptions through its history, the most recent examples being the Mývatn (1724‒1729) and Krafla Fires (1975–1984). The Mývatn Fires opened with a steam-driven eruption that produced the Víti crater. A magmatic intrusion has been inferred as the trigger perturbing the geothermal field hosting Víti, but the cause(s) of the explosive response remain uncertain. Here, we present a detailed stratigraphic reconstruction of the breccia erupted from Víti crater, characterize the lithologies involved in the explosions, reconstruct the pre-eruptive setting, fingerprint the eruption trigger and source depth, and reveal the eruption mechanisms. Our results suggest that the Víti eruption can be classified as a magmatic-hydrothermal type and that it was a complex event with three eruption phases. The injection of rhyolite below a pre-existing convecting hydrothermal system likely triggered the Víti eruption. Heating and pressurization of shallow geothermal fluid initiated disruption of a scoria cone “cap” via an initial series of small explosions involving a pre-existing altered weak zone, with ejection of fragments from at least 60-m depth. This event was superseded by larger, broader, and dominantly shallow explosions (~ 200 m depth) driven by decompression of hydrothermal fluids within highly porous, poorly compacted tuffaceous hyaloclastite. This second phase was triggered when pressurized fluids broke through the scoria cone complex “cap”. At the same time, deep-rooted explosions (~ 1-km depth) began to feed the eruption with large inputs of fragmented rhyolitic juvenile and host rock from a deeper zone. Shallow explosions enlarging the crater dominated the final phase. Our results indicate that at Krafla, as in similar geological contexts, shallow and thin hyaloclastite sequences hosting hot geothermal fluids and capped by low-permeability lithologies (e.g. altered scoria cone complex and/or massive, thick lava flow sequence) are susceptible to explosive failure in the case of shallow magmatic intrusion(s).

**Supplementary Information:**

The online version contains supplementary material available at 10.1007/s00445-021-01502-y.

## Introduction

The Krafla central volcano with its volcanic history (Sæmundsson 1991; Thordarson and Larsen 2007) and geothermal resources (Langella et al. 2017) is the most studied volcano in Iceland. The onset of the Krafla Fires (1975–1984) inspired intensive volcanological research that has greatly increased our understanding of volcanism in extensional rift settings (Einarsson 1991; Thordarson and Larsen 2007; Hjartardóttir et al. 2012; Árnason 2020). Krafla volcano has been closely monitored since the Krafla Fires (Ármannsson et al. 1989), with geothermal exploration and drilling yielding extensive knowledge on the volcano and its hydrothermal systems (Weisenberger et al. 2015 and reference within). Initial geothermal exploration was conducted in 1969 and continued the following years (Ármannsson et al. 1987) resulting in the construction of one of the major geothermal power plants (60 MWe) in Iceland, concomitant with the Krafla Fires that started in December 1975.

The Krafla Fires included a series of discrete basaltic fissure eruptions produced by repeated dike injections within the caldera and were accompanied by intense degassing in the vicinity of the Víti and Hveragil areas (Ármannsson et al. 1989; Einarsson 1991). The Mývatn Fires, which occurred during the period 1724‒1729, were similarly characterized by a series of effusive fissure eruptions within the Krafla caldera volcano and extending along the fissure swarm (Sæmundsson 1991). Despite their similar eruptive styles the Mývatn Fires were distinguished by the Víti explosive eruption at the onset of the rifting episode (Grönvold 1984). The Víti crater has previously been reported by Jónasson (1994) to have been formed by a (steam-driven) “phreatic” explosive event, whereas Rooyakkers et al. (2021) suggest that it was generated by a mixed hydrothermal-magmatic eruption. Víti ejecta includes mostly altered country rocks, with minor aphanitic rhyolitic and basaltic pumices implying a magmatic contribution, as well as felsic and gabbroic lithics indicating the involvement of intrusive rocks at depth. Rhyolites are well known within Krafla, as well as other central volcanoes in Iceland, as was dramatically confirmed by an unexpected encounter with rhyolite magma during the Iceland Deep Drilling Project (IDDP-1) in 2009 (Zierenberg et al. 2013), as well as from other boreholes suspected to have tapped into silicic magma (Hólmgeirsson et al. 2010). Such findings indicate that Krafla caldera is indeed a “basalt-fired crucible” for making rhyolite (Eichelberger 2016), whose storage depth is limited to approximately 2 km, within thick sequences of basaltic dikes and other minor felsic bodies (Mortensen et al. 2014). Examination of the Krafla Fires and several earlier basaltic eruptions of the last 3000 years suggest that they did not disturb the rhyolitic magma and/or the shallow hydrothermal systems at Krafla, in stark contrast with the Víti case. While it appears clear that a rhyolite intrusion caused a perturbation within the geothermal field hosting Víti, the cause(s) of the explosive response remain unclear.

Recent investigations demonstrate how characteristic lithologies and particle properties within steam-driven (phreatic and hydrothermal) eruption deposits, together with the characterization of local geology and alteration settings, can be used to infer the triggers, dynamics, and sources of such eruptions (Breard et al. 2014; Mayer et al. 2015; Montanaro et al. 2016a, 2020; Pittari et al. 2016; D’Elia et al. 2020). Valuable insights into the properties of source rocks can also come from analysing drill cuttings and/or cores from nearby eruptive sites (Mastin 1991; Gallagher et al. 2020). Geothermal wells may also provide direct information on the pressure–temperature profile of the hydrothermal system and on rock properties such as porosity, permeability, and alteration state, thus giving further insight into pre-eruptive settings (Dobson et al. 2003; Mielke et al. 2015; Milicich et al. 2016, 2020; Carlino et al. 2018).

Here, we examine the Víti breccia and ejected particles (blocks and matrix), as well as the exposed lithologies and samples of drill-cuttings adjacent to the explosion site to (1) define a detailed stratigraphy of the breccia deposits, (2) shed light on the pre-eruptive settings and the eruption trigger, and (3) explore relationships between eruption dynamics and host-rock properties during the explosive events that formed Víti crater.

## Geological setting

**Fig. 1:**
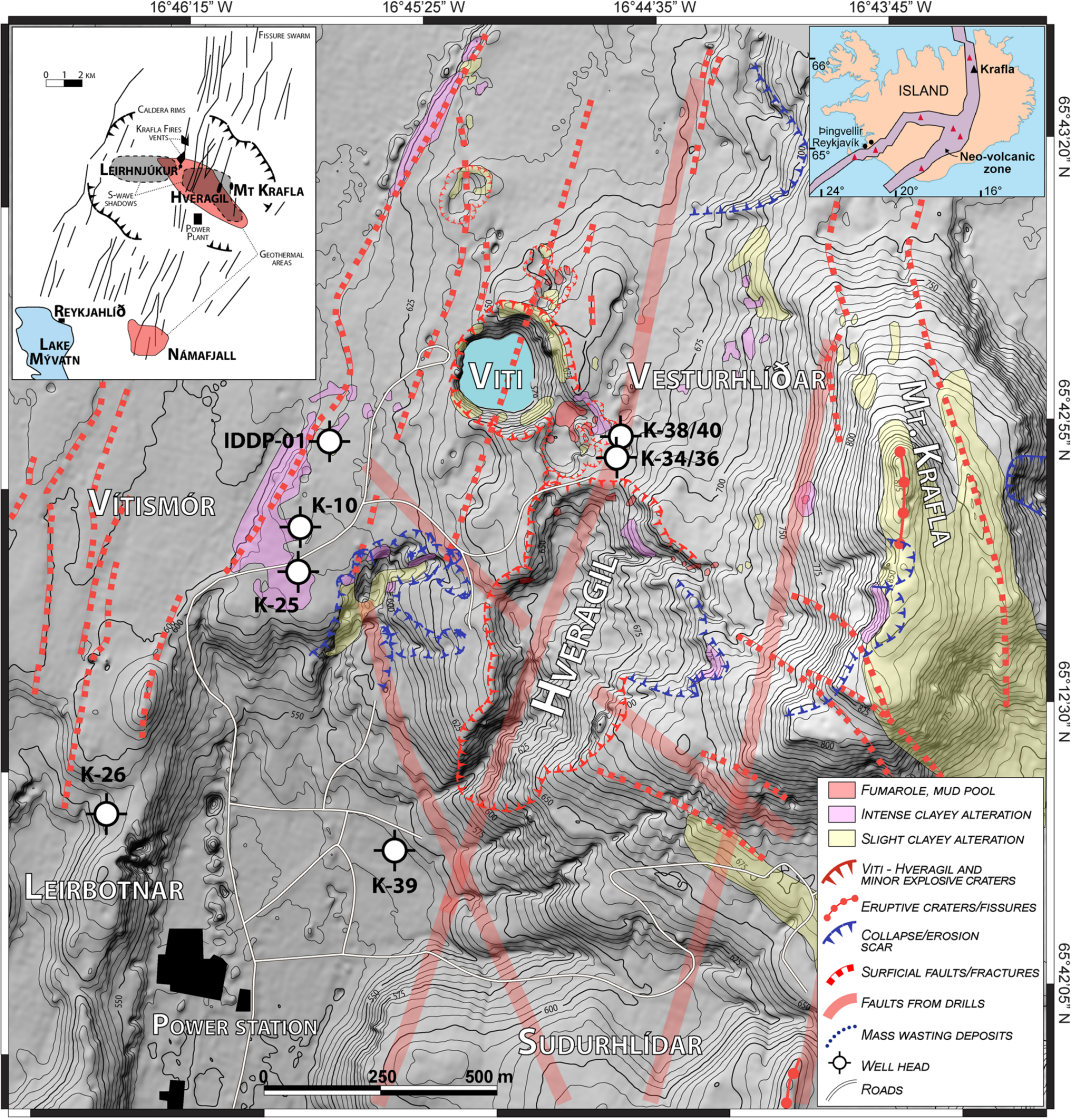
Lidar map (from ArticDEM; Porter et al., 2018) of the Víti crater and Mt. Krafla showing: (i) active geothermal areas (yellow, pink, and red patches), (ii) several rift-related faults, (iii) main volcanic features (explosive craters, fissures, lava deposits), and (iv) mass wasting-related morphological elements. The location of wells IDDP-01, K-10, K-25, K-26, K-34, K-36, K-38, K-39, and K-40 as well as that of the main geothermal fields (Leirbotnar, Vítismór, Suðurhlíðar, Vesturhlíðar) and the power station, is shown (modified from Weisenberger et al., 2015). In the inset: map of Iceland’s most active volcanic zones (shaded purple) and location of Krafla central volcano (black triangle) (right); structural map of Krafla caldera with its active fissure swarms, main geothermal areas, and S-wave shadow zones (left)

The Víti crater is one of the major explosive features, together with Hveragil, located on the western slope of Mt. Krafla, within the caldera of the same name (Fig. 1). The ~ 8 × 10 km caldera (W–E elongated) within the Krafla volcanic system is infilled by basaltic lavas hyaloclastites with minor silicic bodies (Ármannsson et al. 1987) and is bisected by a NNE-SSW trending fissure swarm (Sæmundsson 1991; Hjartardóttir et al. 2012). The Krafla caldera developed from an explosive eruption producing dacitic welded tuff at ca. 110 ka (Calderone et al. 1990; Rooyakkers et al. 2020) and has since remained active with recurring volcanic episodes characterized by predominantly basaltic fissure eruptions and dike injections into the fissure swarm, as well as by intermittent eruptions of intermediate to silicic magma (Sæmundsson 1991, 2008). Such alternating volcanic activity is linked to a bimodal behaviour in tectonics and crustal spreading. At about 8 ka, the spreading moved from the eastern part of the fissure swarm to the western part without any significant increase in volcanic activity and then shifted back again at about 3 ka to produce intense extrusive volcanism (Sæmundsson 1991). A mature geothermal system never developed in the western part of the caldera, whereas high-temperature geothermal systems with extensive surface manifestations developed in the eastern part (Sæmundsson 1991, 2008). These tectonic and volcanic activities are related to different local crustal-spreading directions south (E 22°S) and north (E 4°S) of the caldera that produce a N–S opening component (18°) in the eastern part of the volcano equal to ~ 1/3 of the spreading motion (Árnason 2020). This opening component favoured the ascent of mantle-derived magma and volcanism manifested by subglacial extrusions (e.g. Mt. Krafla), abundant explosion craters in the western slopes of Mt. Krafla (e.g. Víti and Hveragil), as well as by post-glacial eruptions and dike injections centred at Leirhnjúkur (Fig. 1). Additionally, the fracturing process likely greatly enhanced permeability in the shallow rocks west of Hveragil, which are dominated by hyaloclastite and lava deposits. High permeability supported a vigorously convecting, isothermal, system, e.g. at Leirbotnar and Vítismór (Fig. 1; Weisenberger et al. 2015; Árnason 2020; Eggertsson et al. 2020).

Resistivity surveys further show that a ESE-WNW low-density structure separates the highly active northern from the less intense southern intra-caldera geothermal systems (Johnsen 1995; Gasperikova et al. 2015; Rosenkjaer et al. 2015; Magnússon 2016; Scott et al. 2019). Hydrogen isotope ratios in geothermal fluids further indicate different isotopic signatures caused by boiling processes in the northern system (Pope et al. 2016).

Silicic magma encountered in wells K-39 and IDDP-1 at ~ 2-km depth (Hólmgeirsson et al. 2010; Mortensen et al. 2010) suggests that rhyolitic melt, originated by basaltic intrusions into shallow hydrothermally altered basalt, is supplying heat to the geothermal systems above (Elders et al. 2011). Moreover, the presence of such shallow rhyolitic bodies is thought to explain the occasional silicic (often explosive) eruptions that are triggered when substantial amounts of silicic magma are perturbed by major basalt intrusions (Jónasson 1994; Rooyakkers et al. 2021).

### Recent eruptions and geothermal manifestations at Krafla

**Fig. 2:**
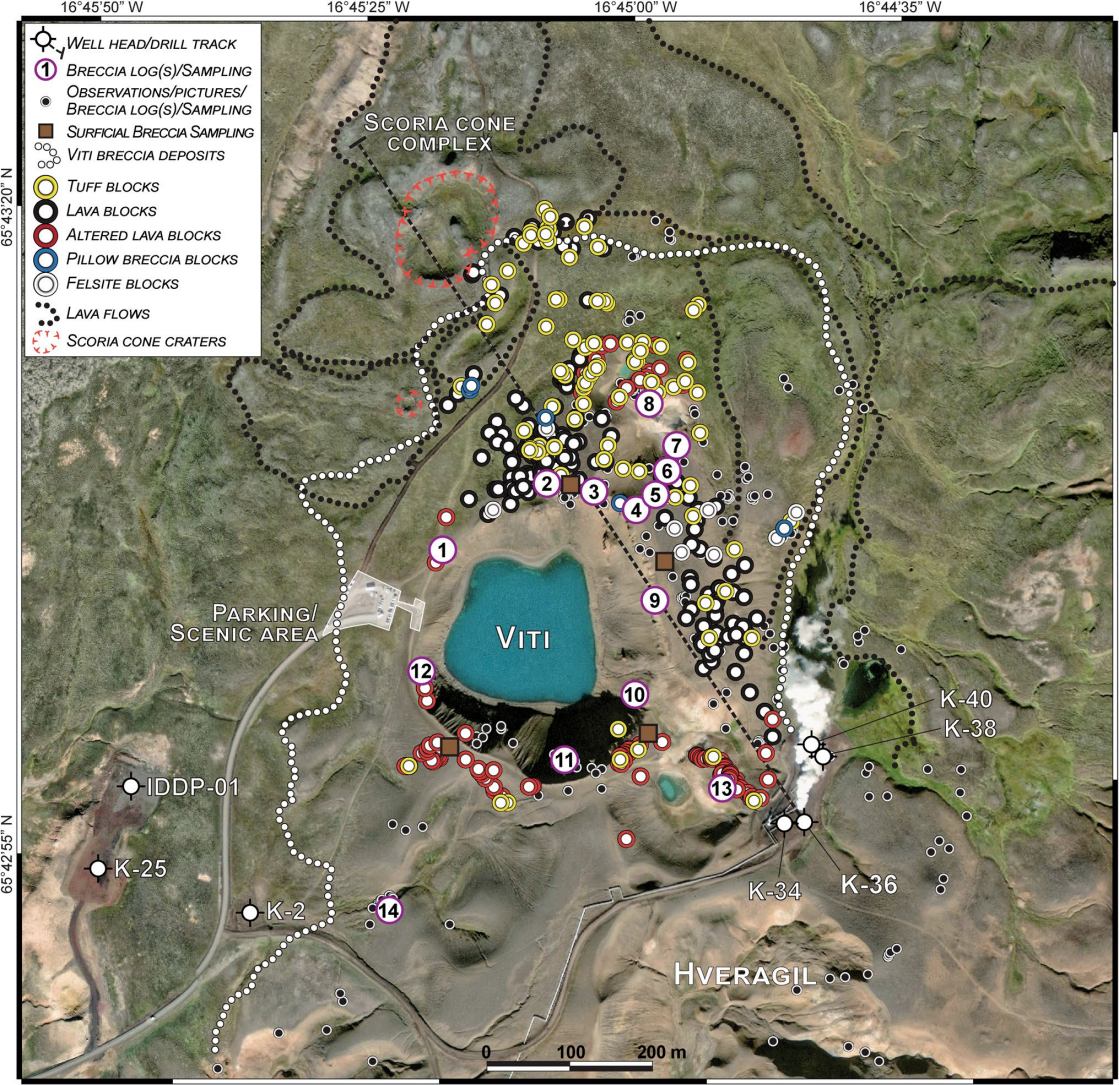
Satellite image (Google Earth™, 2016) of Víti crater and surrounding areas, showing the investigated and sampled areas. The dotted white line indicates the limit of the breccia deposits produced during the Víti eruption. Numbered circles indicate main stratigraphic logs, while the position of coarse ballistic blocks (> 25 cm) is shown by coloured circles distinguished per lithology (tuffs, lavas, pillow breccia, felsite). Lava flow bodies and scoria cone craters in the surroundings of Víti are indicated. Locations of wells IDDP-01, K-25, K-2, K-34, K-36, K-38, and K-40 are shown, as well as the drill track of well K-36 that was drilled in the close proximity of the eruption site (dashed black line). Tourist parking and scenic area are also indicated (transparent white area)

During the past 3000 years, eruptions in Krafla have taken place every 300–1000 years (Sæmundsson 1991). The two latest eruptive phases of the Krafla volcano were the Mývatn (1724‒1729) and Krafla Fires (1975‒1984). The Mývatn Fires started on May 1724 with the Víti eruption forming a subcircular, 350 × 280 m crater, and a cluster of 50‒70-m-wide craters located NNE of the main vent (Figs. 1 and 2). The Víti eruption was followed by repeated dike injections into the fissure swarm, centred at Leirhnjúkur (Fig. 1). Following, two basaltic fissure eruptions took place in 1727 and 1729 mainly within the caldera (Sæmundsson 1991). The Krafla Fires started with a small basaltic fissure eruption within the caldera, followed by repeated dike injections and occasional small eruptions until 1980, while four main fissure eruptions took place from 1981 to 1984 (Sæmundsson 1991). Modelling of the deformation that occurred during and after a Krafla Fires eruption has indicated a centre of inflation/deflation at depths of 3.9–7.5 km, and multiple magma reservoirs down to 3-km depth (Tryggvason 1986; Arnadottir et al. 1998)—an interpretation that is consistent with a previous study that recognized the presence of a less dense voluminous body attenuating S-waves interpreted to contain magma that extends from ~ 3 to ~ 7-km depth (Fig. 1; Einarsson 1978).

Surface activity in the geothermal area at Krafla in terms of hot springs, fumaroles, and geothermal alteration distribution generally lies above the inferred magma chamber. Geothermal features and their spatial evolution were closely monitored during the Krafla Fires, and since the initial geothermal resource assessment of the Krafla area (Weisenberger et al. 2015 and reference therein). The geothermal activity has been mainly concentrated on the western and southern slopes of Mt. Krafla and at Leirhnjúkur in the centre of the caldera (Fig. 1). During the Mývatn and Krafla Fires, large scale faulting extended north and south from these areas and intersected volcanic eruption sites; some of the normal faults displaced the southern slope of Mt. Krafla where fumaroles became active. Generally, the degassing activity increased significantly during both volcanic episodes with large CO_2_ emissions in Leirhnjúkur, Hveragil, and Víti areas (Ármannsson et al. 1989, 2013). Particularly, from the onset and during the Krafla Fires, widespread fumaroles and mud pools developed in the Hveragil fissure, as well as around the Víti crater and along surficial active faults. Geothermal activity generally subsided within the following 30 years, with only a few fumaroles and mud pools remaining (Fig. 1; Ármannsson et al. 1987, 2013; Weisenberger et al. 2015).

Diffuse CO_2_ degassing surveys carried between 2004 and 2008 detected a significant flux in Suðurhlíðar, Vesturhlíðar, and Leirhnjúkur areas, with lower levels in Leirbotnar and Vítismór (Fig. 1; Ármannsson et al. 2007; Dereinda 2010; Ármannsson 2017). Emissions to the north of Víti and south of Suðurhlíðar are barely detectable, whereas gas emission anomalies within this boundary indicate that fluids travel upward to the surface mainly along NNE-directed fractures, with secondary discharges along WNW trending fissures (Fig. 1; Dereinda 2010).

The input of magmatic CO_2_ resulted in intense reactions with the Ca-rich basaltic glass and feldspar and in the precipitation of copious amounts of calcite, which is found in Krafla drill cuttings and in surficial deposits (Gudmundsson and Arnórsson 2005; Thien et al. 2015; Weisenberger et al. 2015). In Leirbotnar, calcite formation reaches greater depths than at Suðurhlíðar and Vesturhlíðar (Ármannsson et al. 2007), consistent with the notion that calcite formation in the region is dominated by colder fluids, rather than occurring in hotter areas where calcite is replaced by wollastonite at > 290‒300 °C.

## Field study and well stratigraphy

**Fig. 3:**
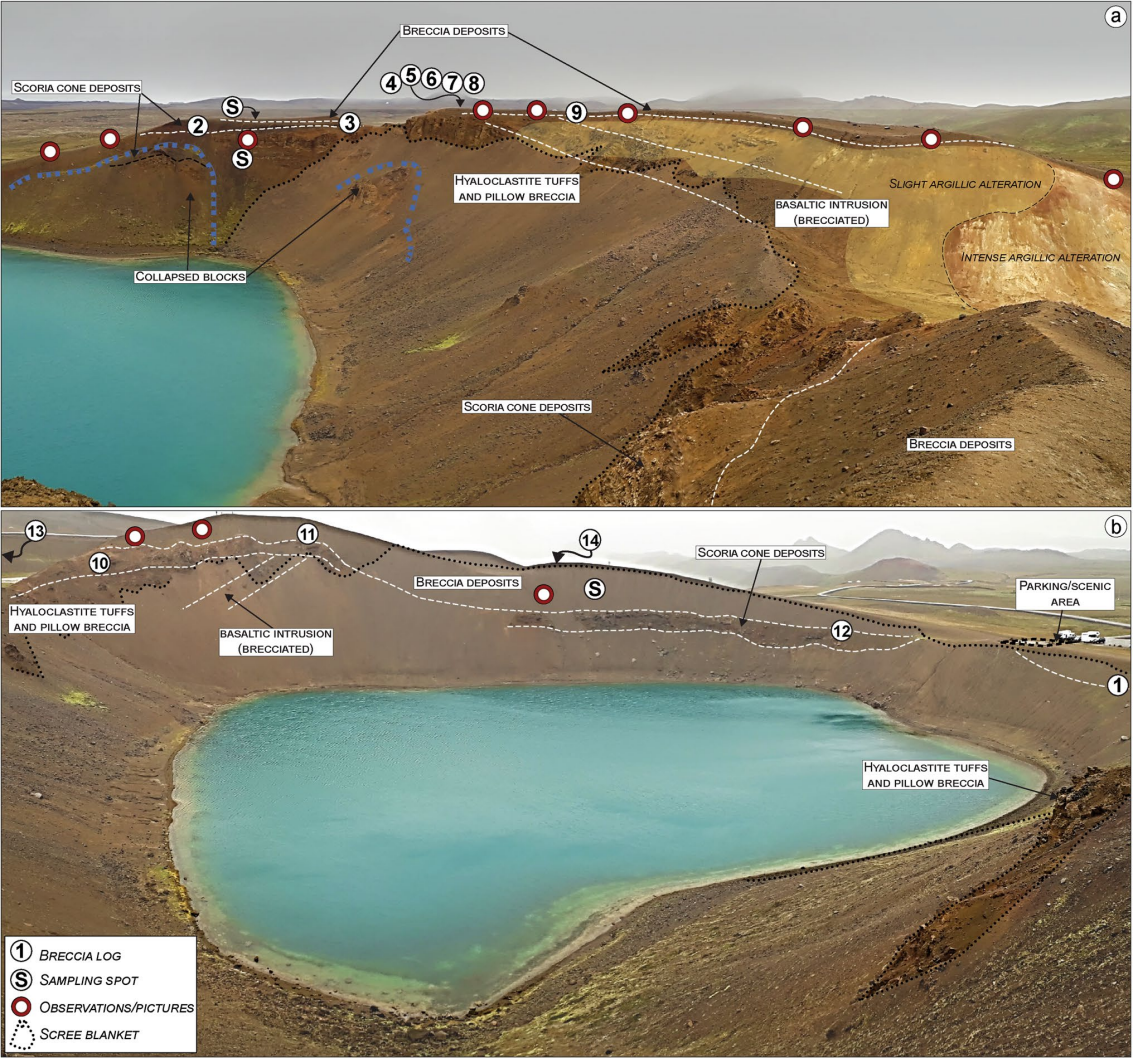
View of northern and southern sides of Víti lake showing the main outcropping lithologies, altered areas, and geomorphological elements (i.e. collapsed blocks), as well as the sampling and observation locations. **a** North-to-north-eastward view: a ca. 40-m-thick hyaloclastite sequence forms the dominant lithological bedrock overlain by a scoria cone deposit on the north side, and cut by a basaltic intrusion on the northeastern side. Atop these sequences, a series of breccia deposits up to 2-m thick can be found. The northeastern side also shows zones of slight-to-intense argillic alteration. **b** In the southern side, a 10 to 30-m-thick outcropping hyaloclastite is covered by a meters-thick series of lava flow (part of a scoria cone deposit), and cut by a basaltic intrusion. The breccia deposits, up to 10-m thick, overlay the lava flows. Notice how most of the inner crater is covered by a scree blanket made mostly of eroded breccia and hyaloclastite

**Fig. 4:**
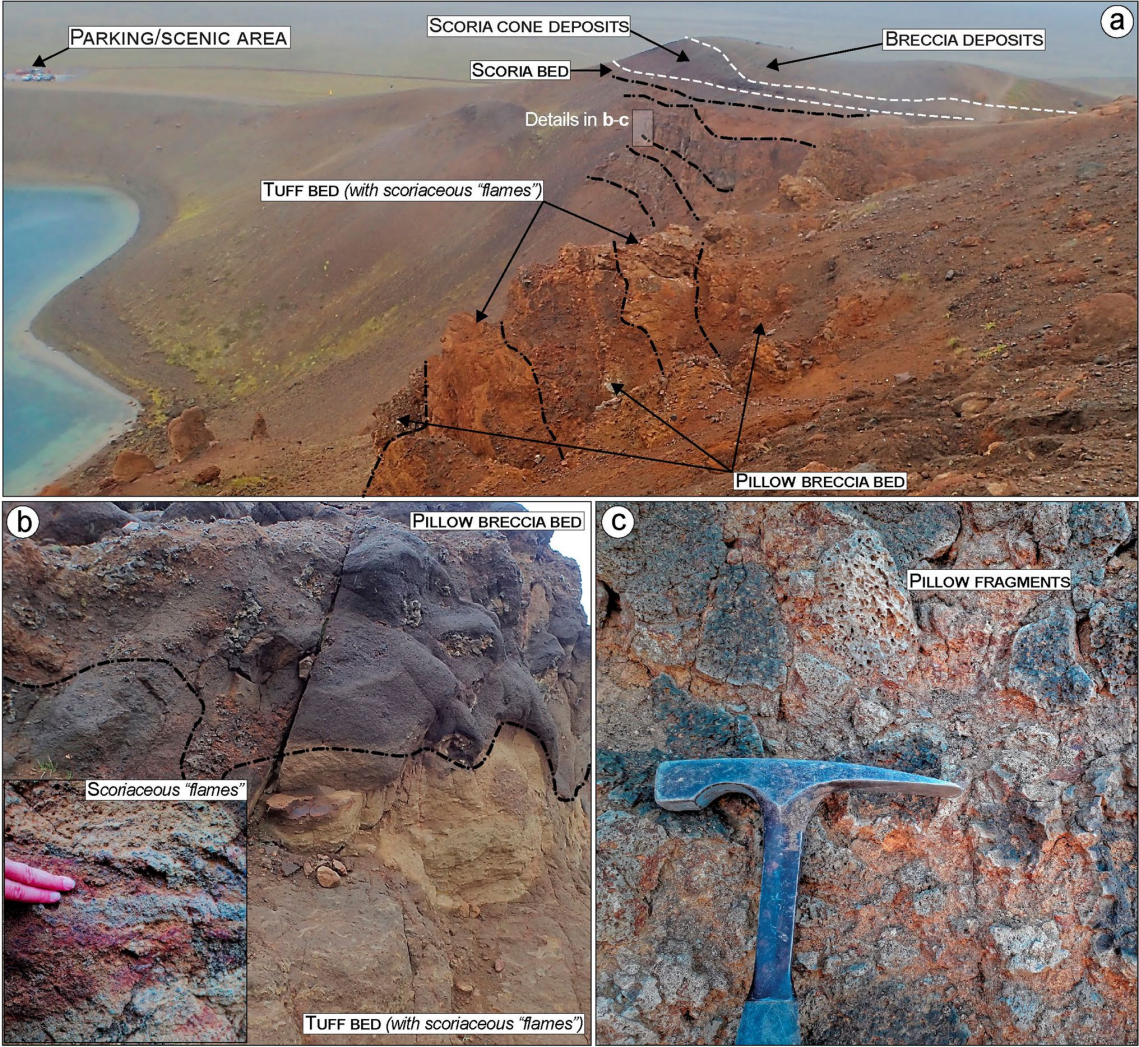
Detailed view of the northern side of Víti lake showing the main outcropping bedrock lithologies. **a** Side view of the ca. 40-m-thick hyaloclastite sequence made of alternating tuffs and pillow breccia beds, overlain by scoria cone deposits in turn covered by breccia deposits. **b** Detailed view of tuffs and breccia showing the typical irregular, erosional contact between the beds. In the inset a close up of scoriaceous “flames” (individual or fragmented scoria welded and deformed). **c** Close up of the pillow breccia showing fragments of pillow in a fine matrix of palagonite

**Fig. 5:**
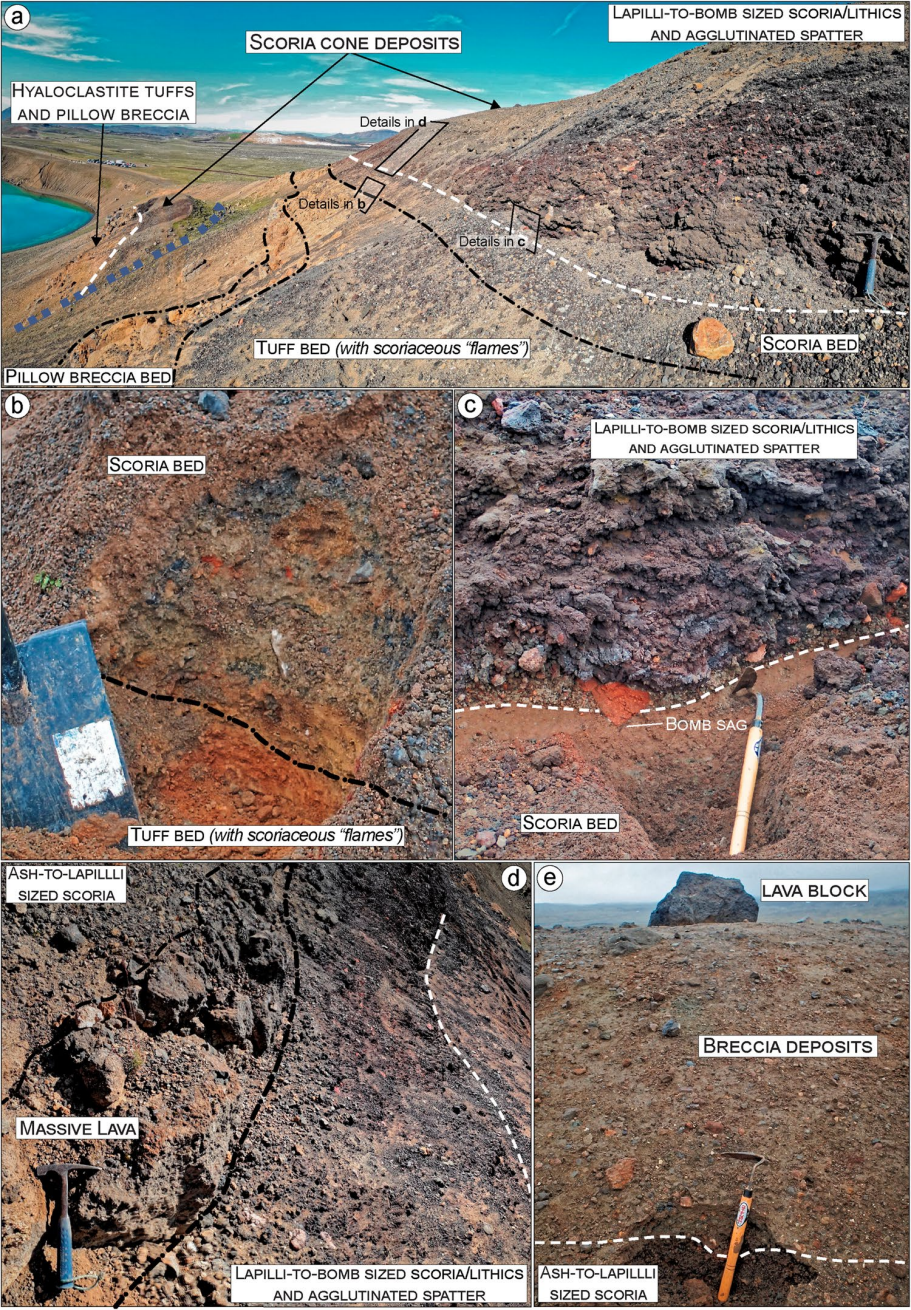
View of northern side of Víti Lake showing the contact between the hyaloclastite unit and the scoria cone deposits. **a** Close view of the top part of the hyaloclastite sequence and the scoria cone deposits. **b** Contact between the scoria-dominated bed, typical of magmatic phases, with the underlying tuff bed. **c** Rare bombs from the scoria cone deposit make sags that penetrate the underlying hyaloclastite scoria bed. **c–d** Scoria cone deposits made of: (i) basal fine-to-coarse ash and lapilli level, rich in altered lithics; (ii) a 1–2-thick spatter scoria, and (iii) ~ 1.5-m alternating lava flow and ash-to-lapilli scoria fall deposits. Collapsed wall blocks show the same hyaloclastite/scoria cone sequence. **e** An ash-to-lapilli scoria level (only the top part of the ~ 0.6-m-thick level is shown) can be found atop the scoria cone deposit sequence (just above the outcrop shown in (**a**)). A sharp contact, locally erosive, separates this level from the overlying breccias. The Nejiri weeder and the hammer are 35-cm long

**Fig. 6:**
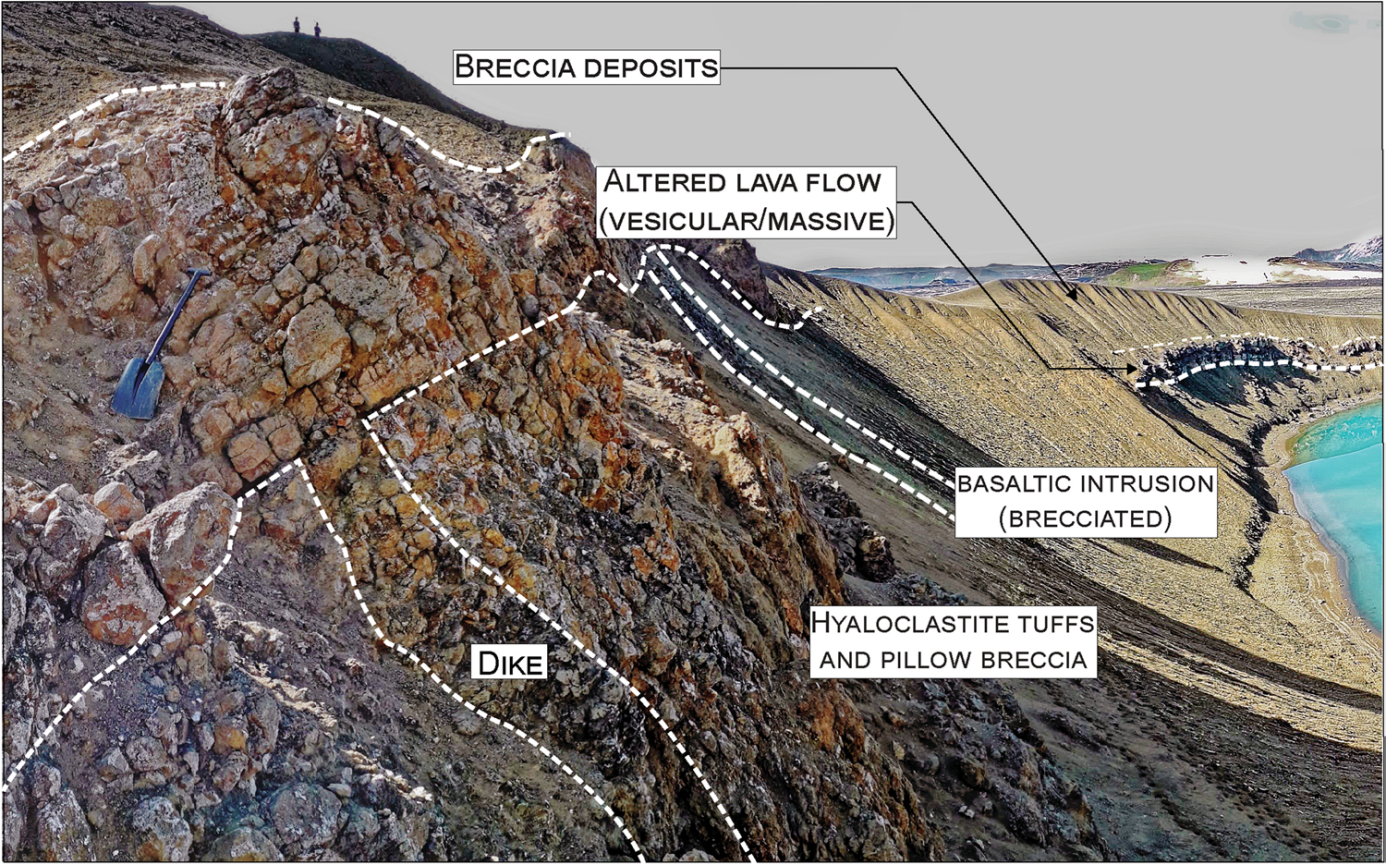
Overview of bedrock lithologies and breccia deposits along the southern crater borders. In the southeastern side (location 10 in Fig. 2), one dike feeding lava flows from the scoria cone complex cuts the hyaloclastite tuff and pillow breccia beds. The basaltic intrusion is also indicated. Breccia deposits cover the lava flows. The spade is 100-cm long

**Fig. 7:**
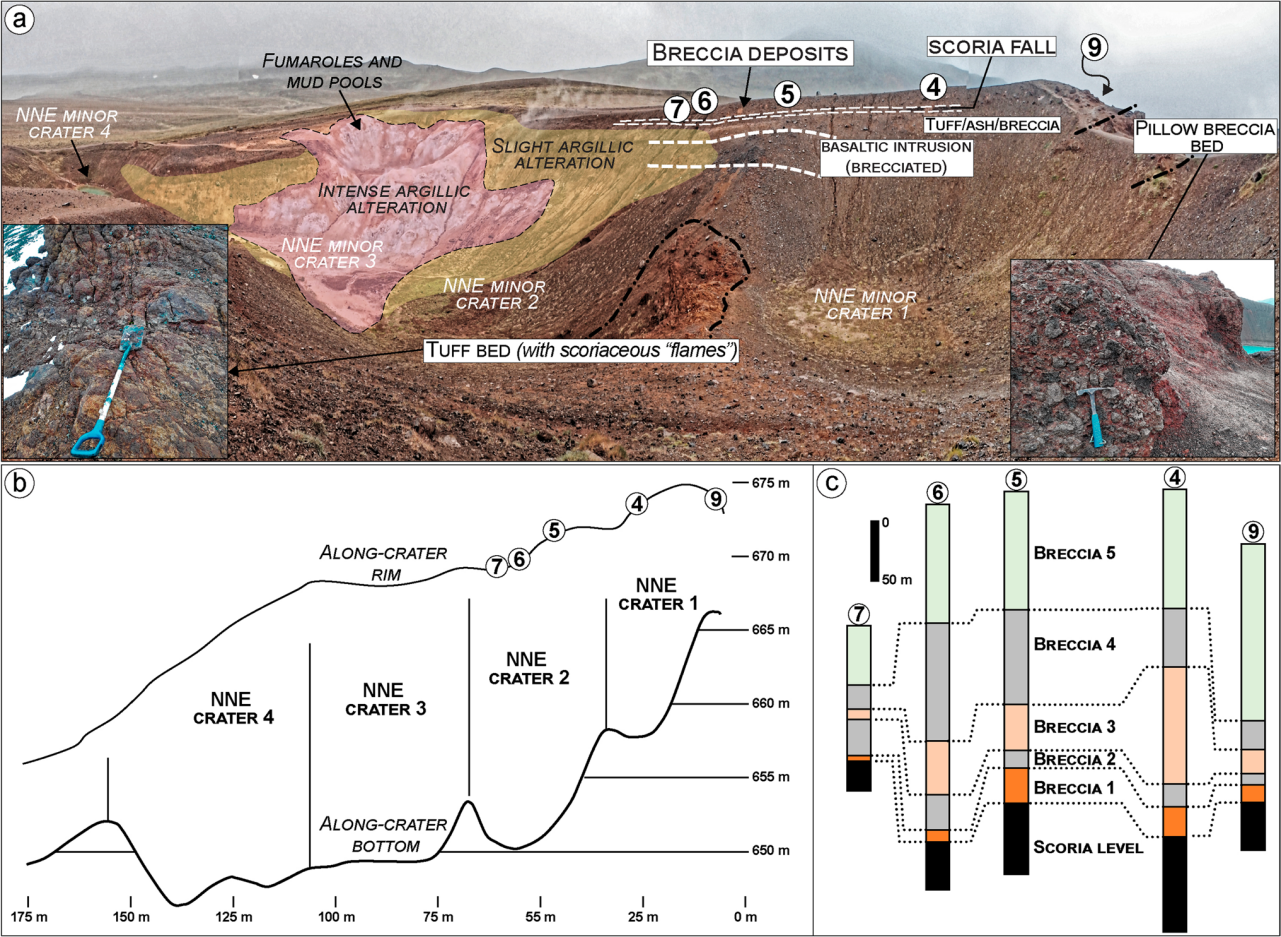
View of secondary crater cluster (1‒4; see Fig. 2) north-northeast of the Víti crater showing bedrock lithologies, breccia deposit distributions, and thickness variations. **a** Crater cluster view seen from the western rim with exposed bedrock including, from bottom to the top: hyaloclastite tuff and pillow breccia; brecciated basaltic intrusion; scoria level from the scoria cone complex (not indicated); tuff/ash levels; older breccia deposit; ash-to-lapilli-sized scoria level; and Víti’s breccia deposits. On the northernmost part, around craters 3 and 4, and partially in and around crater 2, the bedrock units are altered to clays, and fumaroles and mud pools are present. **b** Topographic profiles of both the eastern crater rim and along the depocenter axis showing rim-to-crater-floor depths that range between 15 and 20 m.** c** Víti’s breccia thickness at logs 4‒9: Five breccia deposits can be recognized with thickness variations indicating proximity to the originating crater

**Fig. 8:**
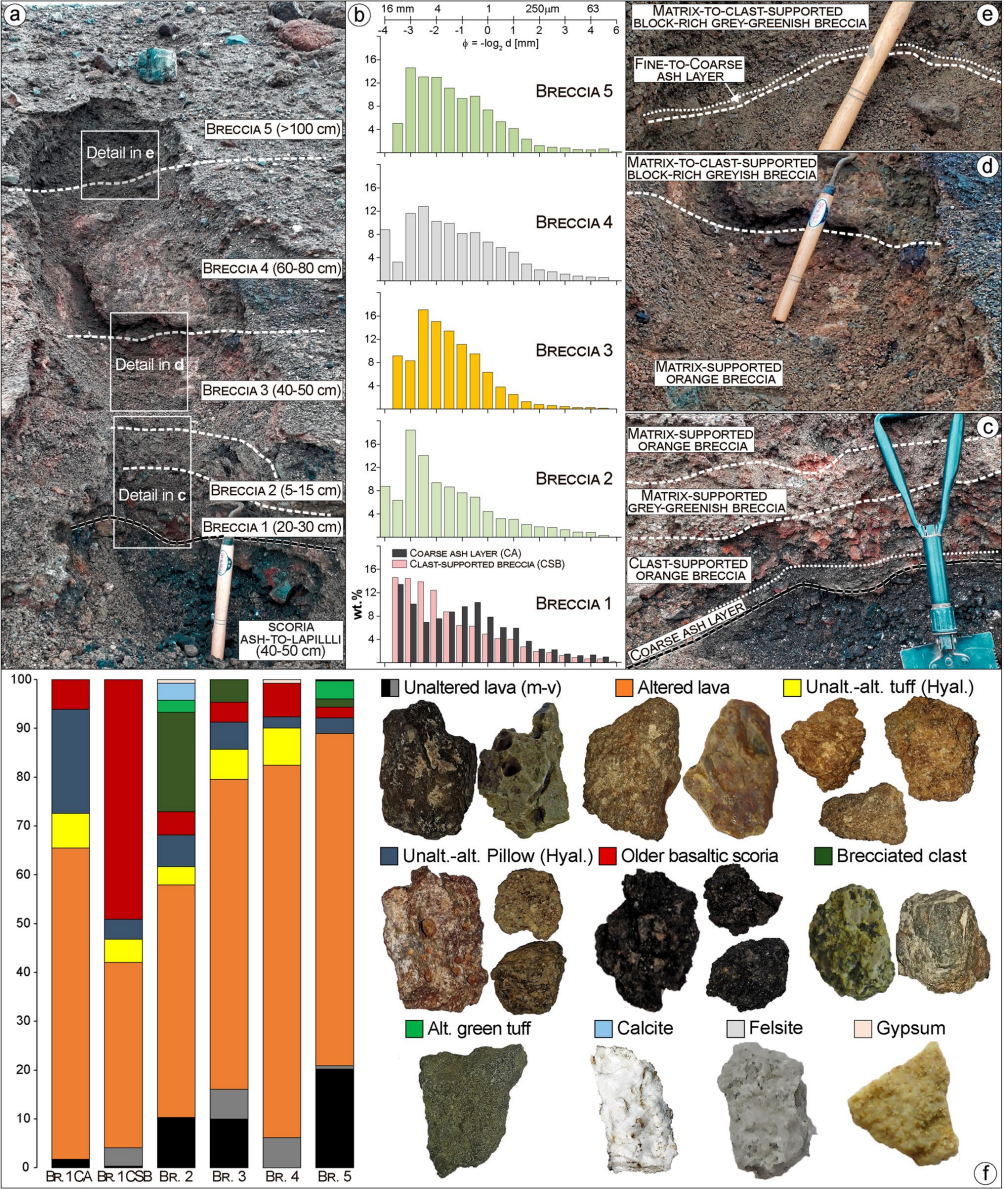
Detailed view of Víti’s breccia deposits outcropping at Sect. 5 (see location in Figs. 2 and 7). **a** Overview of the five breccia deposit; thickness variations (in bracket) are reported for all breccia and scoria fall deposits. **b** Grain size distribution (cumulative weight%) of breccia matrix in the five deposits, and of the coarse ash layer characterizing opening of the eruption. **c–e** Detailed view and textural-colour description of the breccia deposits. The Nejiri weeder and the spade used as scales are 35 and 60-cm long, respectively. **f** Componentry determined from point counts (min. 300 grains) of 1‒8 mm fraction in vol.% and main component specimens

**Fig. 9:**
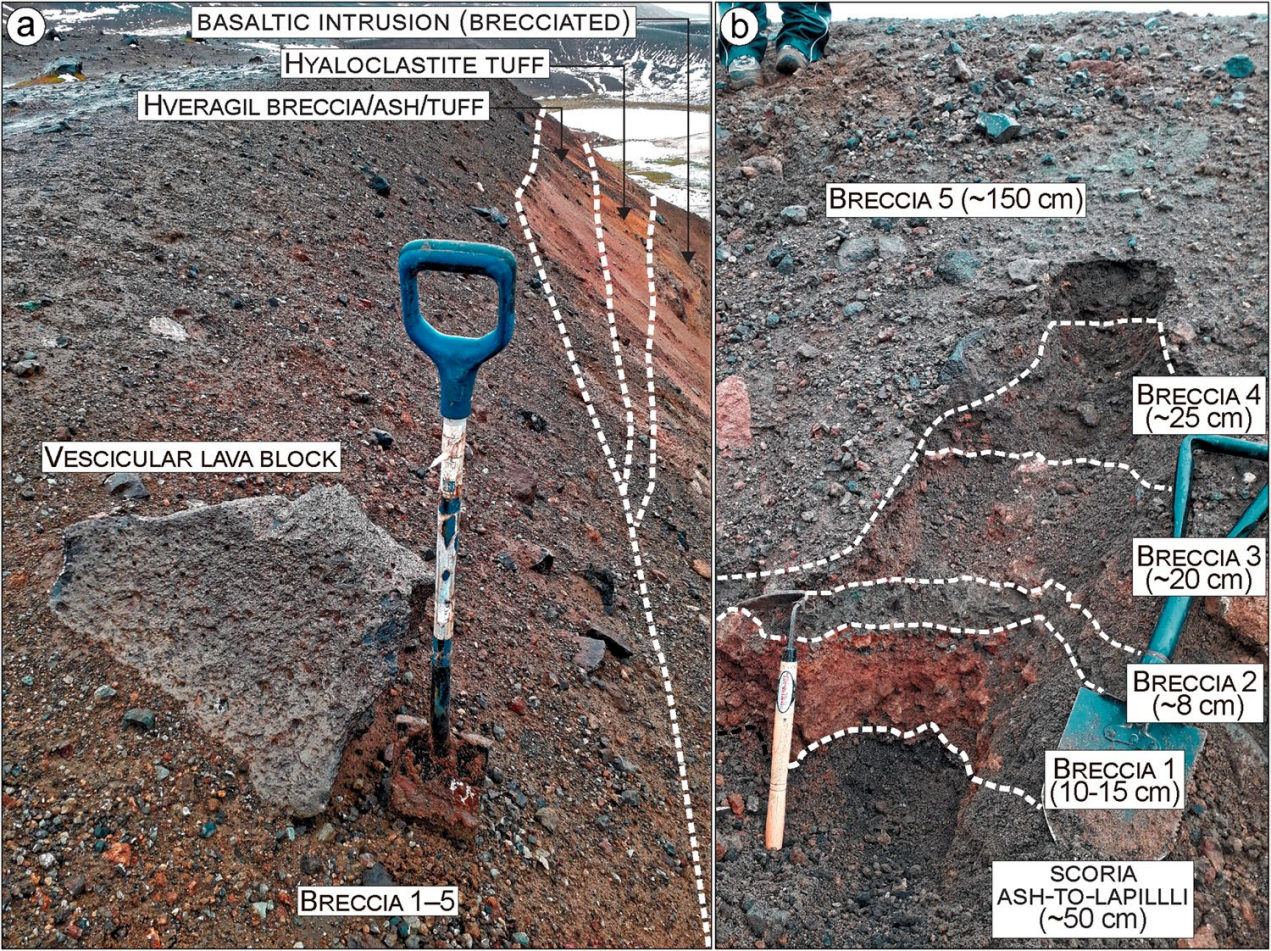
View of the five breccia deposits at Sect. 9 (see location in Figs. 2 and 7). **a** Víti’s breccia deposits overlying the sequence of scoria (shown in (**b**)), Hveragil breccia, ash/tuff deposits, hyaloclastite tuff, and the basaltic intrusion. Large ballistic blocks are associated with the Breccia 5 (the section in **b** is just before the block). **b** Textures, colours, and thickness of the five breccia deposits. Note that the breccias 1‒4 are unconformably overlain by Breccia 5. The Nejiri weeder and the spades used as scale are 35, 60, and 100-cm long, respectively

**Fig. 10:**
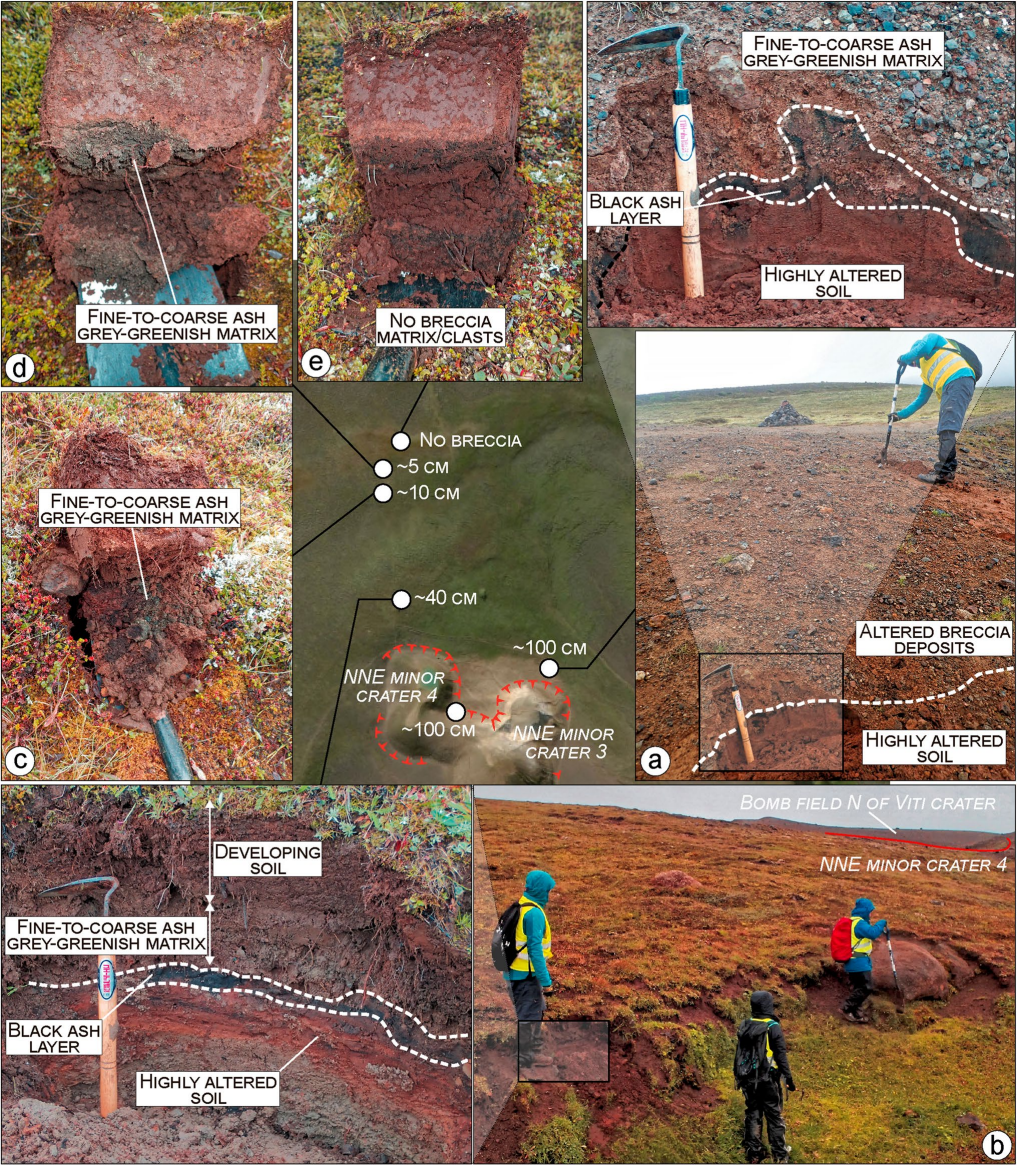
Breccia distribution and thickness variation north of the minor craters cluster. **a** In proximal areas the deposit is ~ 1-m thick, highly altered, and mostly made of breccias 1 and 2, capped by breccia 5. A cm-thick black ash layer separates the Víti breccia deposits from a highly altered and orangish soil (see also (**b**)). **b** The breccia rapidly reduces to 40 cm within a 40-m distance. **c–d** At ~ 120 m, the breccia is found in patches 5–10-cm thick, and at ~ 180 m, no more breccia is found. Far from the craters, most of the breccia deposit is turned into soil and typically shows a grey-greenish remnant of the original matrix. The Nejiri weeder used as scale is 35-cm long

**Fig. 11:**
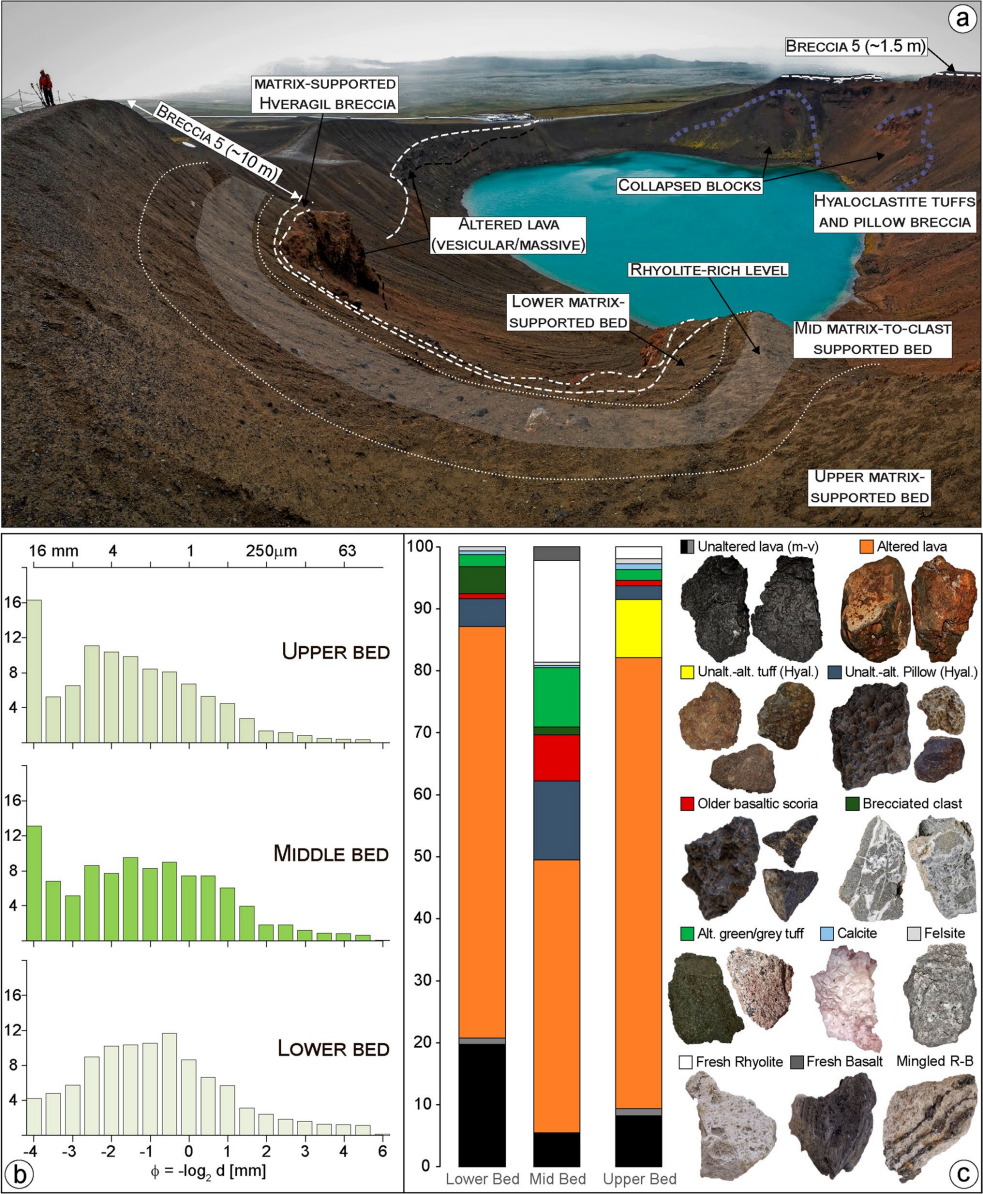
**a** Overview of Víti breccia 5 in the southern sector showing the three main breccia beds and underlying bedrock lithologies; the portion of the central bed rich in fresh rhyolite material is indicated by the transparent white area. Note the difference in the max. proximal thickness of breccia 5, which is up to 10 m in the south, and ~ 1.5 m in the north. **b** Grain size distribution of the three breccia bed matrices. **c** Componentry determined from point counts (min. 300 grains) of 1‒8 mm fraction in vol.% and main components specimens. The mingled rhyolite-basalt (R-B) clasts are rare (< 0.1%) and not reported in the vol.% graph

**Fig. 12:**
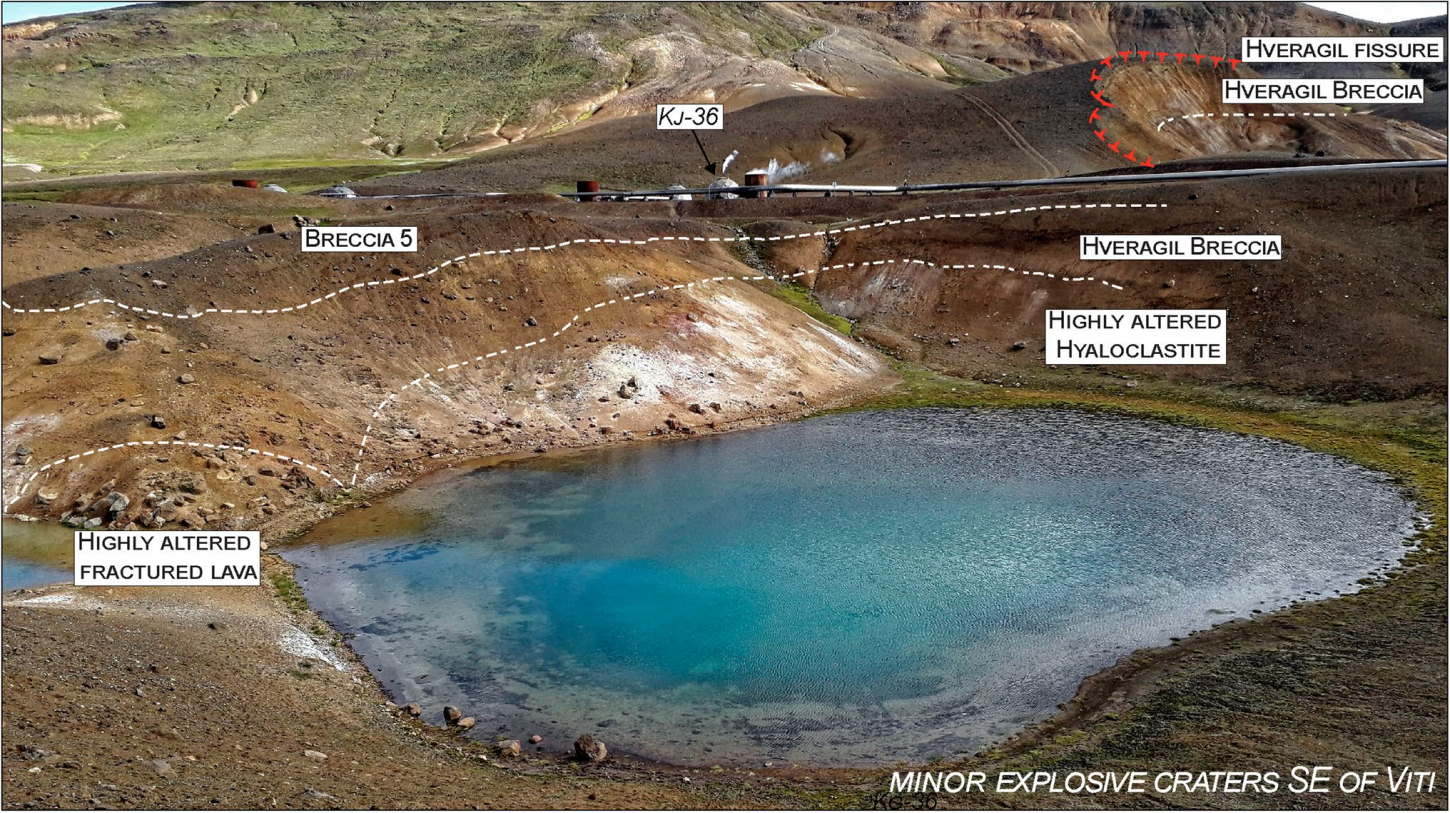
View of a minor explosive crater southeast of Víti, showing bedrock lithologies and the Víti breccia 5. The lower lava and tuff are highly altered, fractured, and covered by a breccia deposit erupted from the close-by Hveragil fissure. Breccia 5 on this side consists of a matrix-to-clast supported deposit, with abundant blocks mostly made of altered lavas and minor tuffs, and overlies directly the Hveragil breccia with an erosional contact. Subsidiary rhyolite lapilli and coarse ash can be found in the deposit

**Fig. 13:**
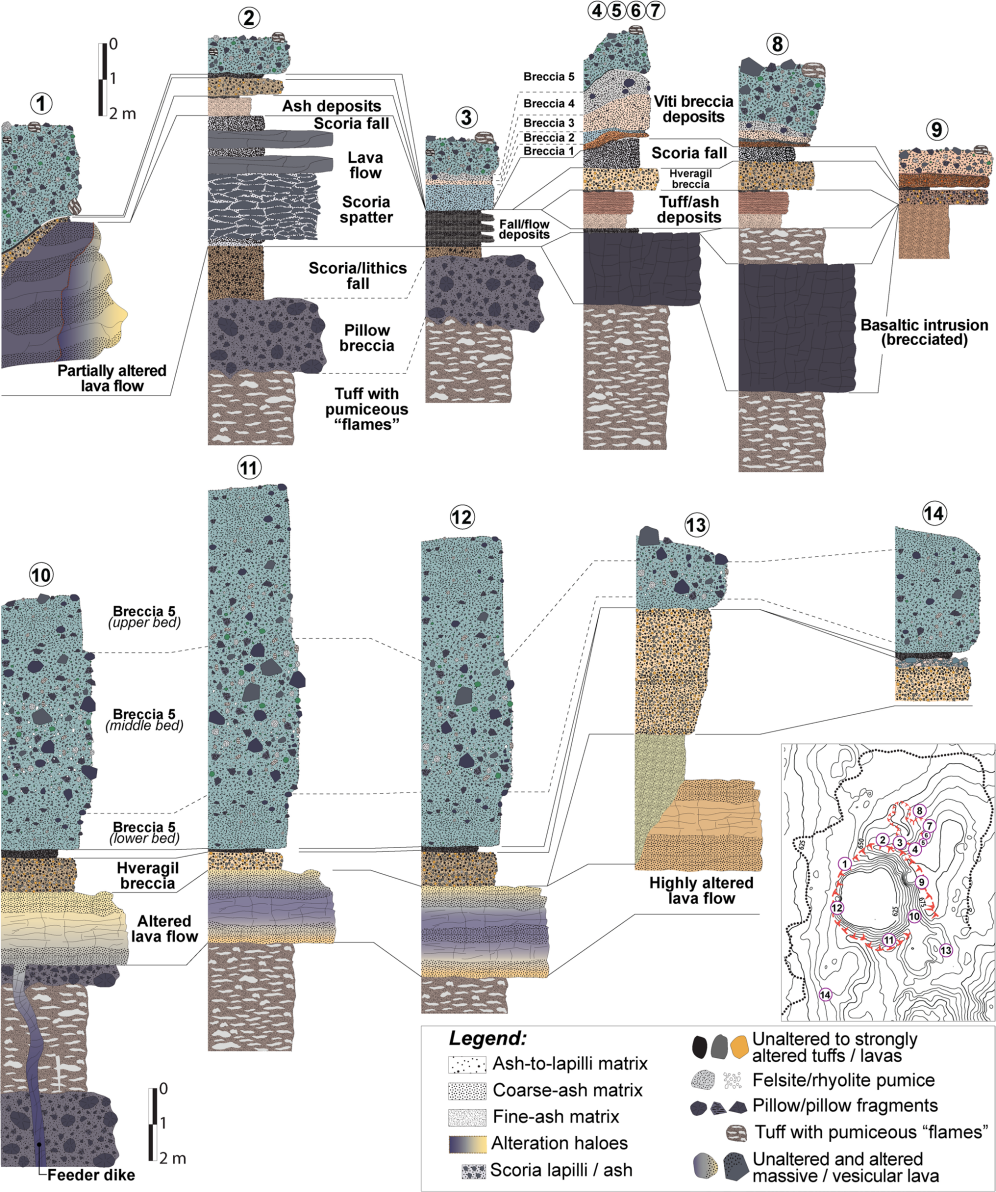
Stratigraphic sections of outcropping breccia, scoria cone, lava flow, and hyaloclastite units surrounding Víti crater. The upper part (1─9) shows the proximal sections from the northern part of the lake, which include bedrock lithologies and breccia erupted from both Víti main crater and the minor crater cluster NNE of the lake. The lower part (10─14) shows the proximal sections from the south and southeastern part of the lake, which include bedrock lithologies and breccia 5. Dashed lines separate beds within the recognized units. For each section, locations are given in the contour map (5-m interval contours) in the lower right, where also the main and minor crater rims, as well as the breccia deposit limit (dotted line), are reported

**Fig. 14:**
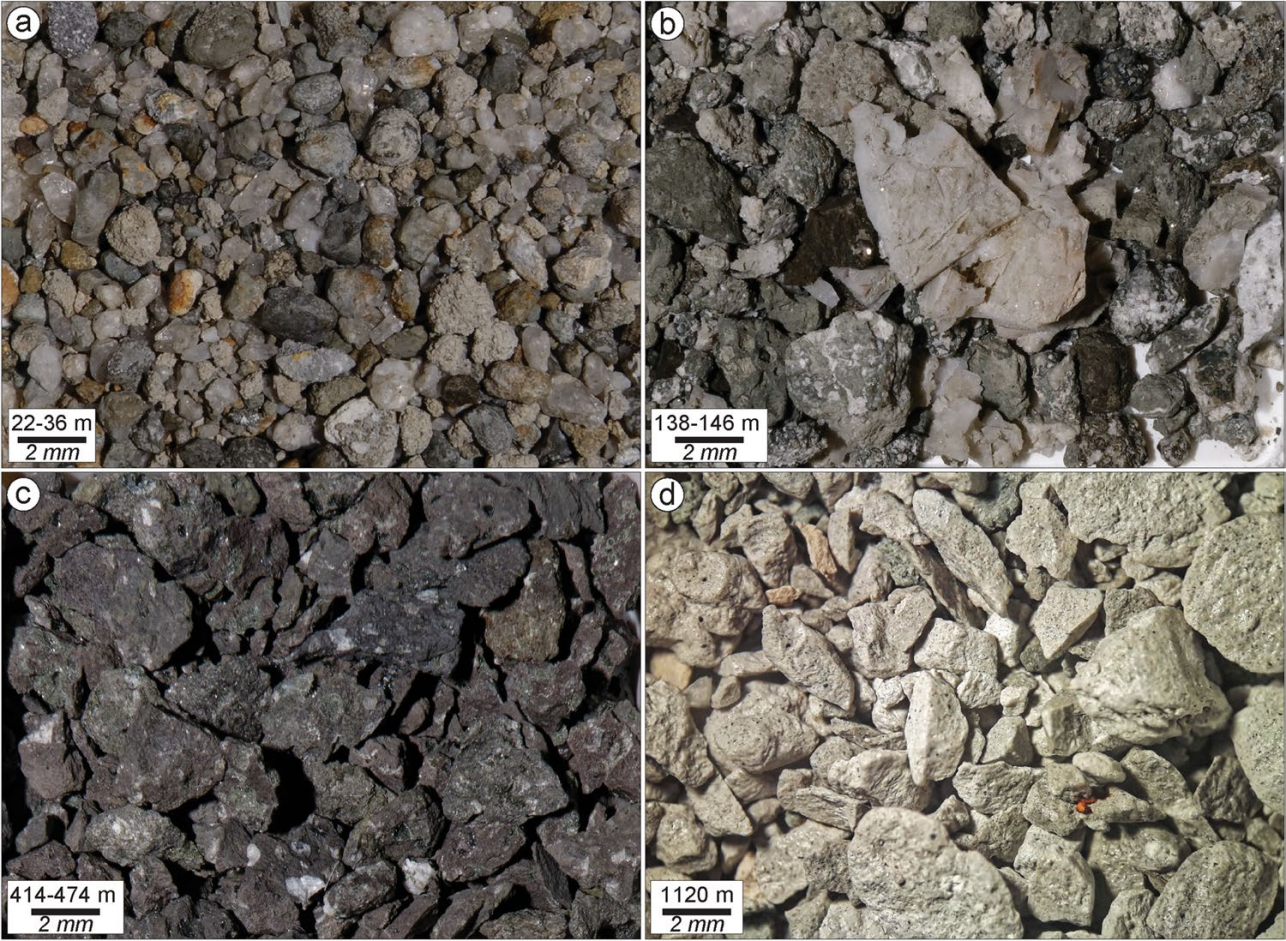
Example of representative lithologies from the main geological units drilled at K-36: **a** from the 14 to 100-m depth, hyaloclastite consisting of mixed aphanitic basalt breccias and tuff; **b** from the 100 to 234-m depth, consolidated and greenish altered basaltic hyaloclastite (fine- and medium-grained tuffs), with abundant calcite; **c** from the 234 to 822-m depth, moderately altered, fine-medium grained basaltic lava with plagioclase phenocrysts; **d** white felsite fragments recovered at 1120 m drilling depth, with no clear evidence of intrusive contact with the host basalt. More data on the drilled lithologies discussed in the text are reported in Fig. ESM 4

**Fig. 15:**
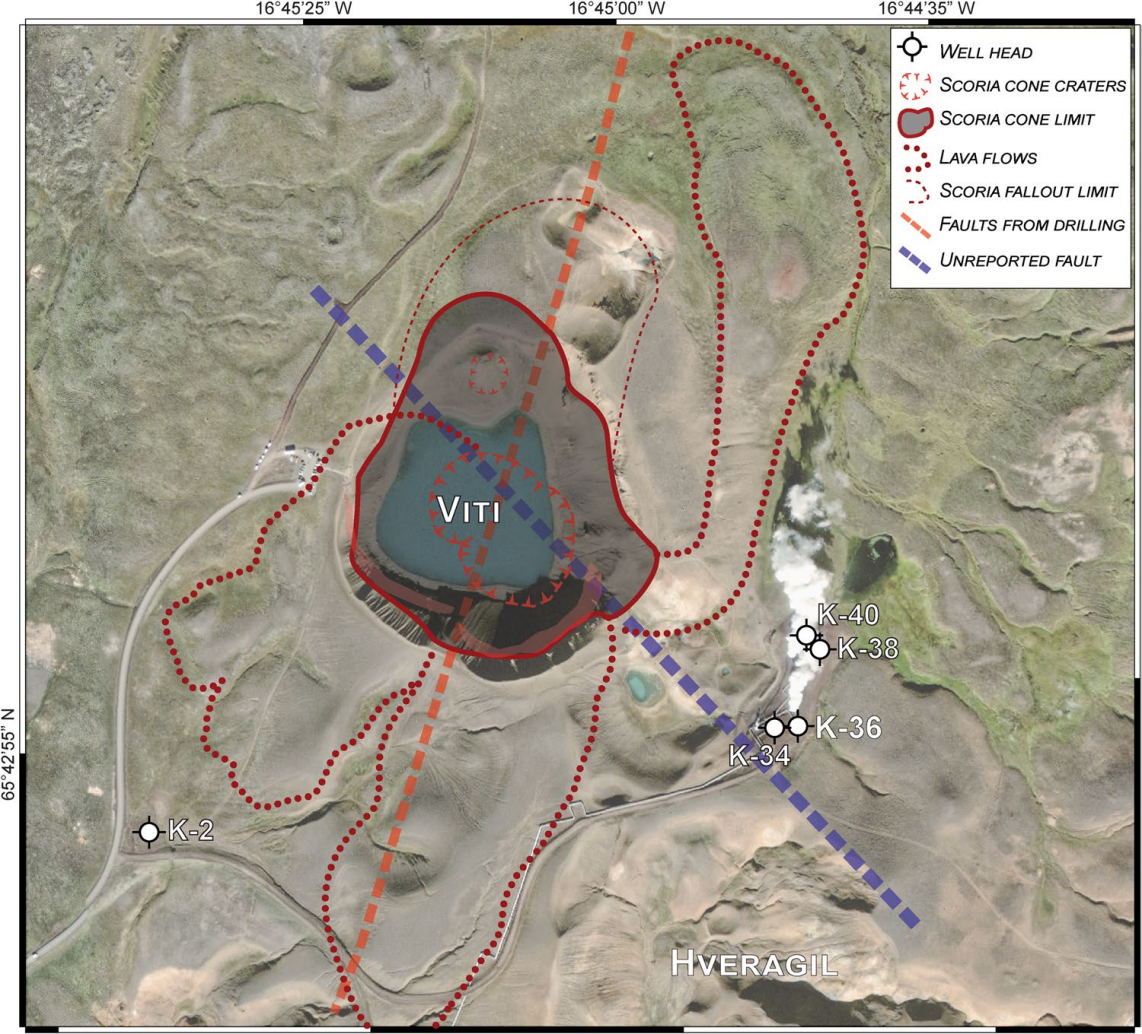
Satellite image (Google Earth™, 2016) of Víti crater and surrounding areas, showing the reconstructed pre-eruption setting that includes the following: (i) the likely extension of the main scoria cone edifice, scoria fallout, and the main lava flow erupted from it; (ii) the NNE-directed fault detected from nearby drills (e.g. K-36, K-34), together with a previously unreported fault. The locations of wells K-2, K-34, K-36, K-38, and K-40 are shown

**Fig. 16:**
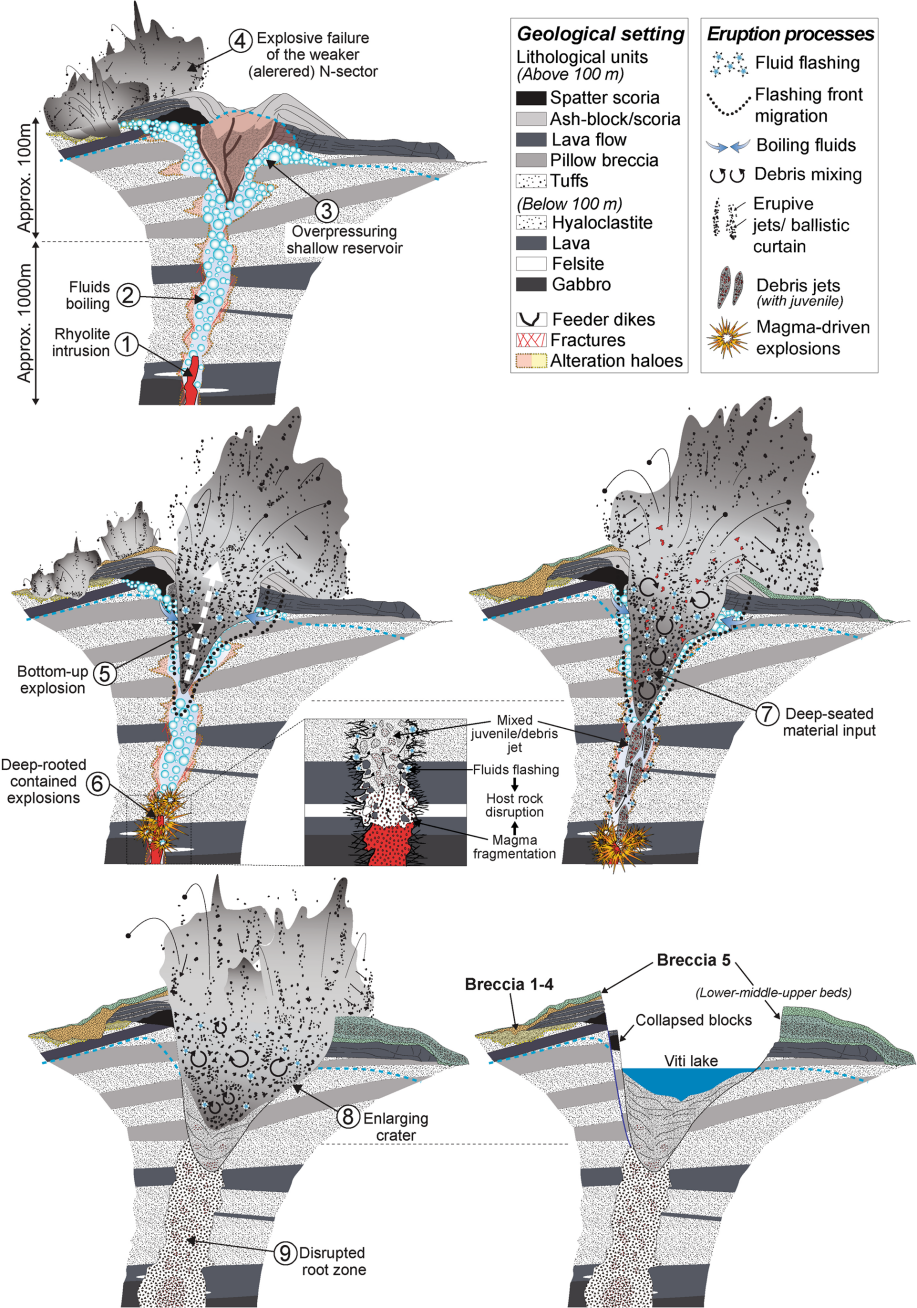
Sketches (not to scale) of the Víti magmatic-hydrothermal eruption’s evolution. The heat pulse provided by a shallow rhyolite intrusion (1) results in boiling of fluids within pores and fractures, pressurizing the shallow part of the hydrothermal system (2–3). An initial series of explosion initiates in the northern sector weakened by alteration (4), while pressure accumulates below the main scoria cone edifice, until there is explosive failure by a bottom-up mechanism (5). At depth, fragmentation of magma and thermal flashing of fluids started a series of deep-rooted explosions disrupting the host rocks (6). Churning mixes fragmented magma and rock debris that are incrementally transported upward to optimal depths and eventually erupted (7). Continuous flashing of fluids likely fuelled shallow explosions and subsequently enlarged the crater (8), while the decrease in intensity (or deepening) of the of deep-rooted explosions resulted in increasingly confined “non-erupting” explosions, with no further contribution of juvenile and deep-seated material (9). Final collapse of the crater rim material lent the crater its final shape

We documented and mapped the distribution of breccia deposits around Víti crater and surrounding minor explosive features, as well as the country rock units involved in the eruption and partially outcropping within the crater’s area (Figs. 2, 3, 4, 5, 6, 7, 8, 9, 10, 11, 12, and 13). We also sampled the ash- and lapilli-rich breccia matrix, and blocks representative of the main lithologies, to be used for component and grain size analyses. Average percentages of each component were analysed from the coarse ash to coarse lapilli range. Distribution and lithology of 350 ballistic blocks > 25 cm was also determined. Examination of macroscopic alteration features (e.g. colour differences), as well as the occurrence of alteration halos and veins, indicated the relative degree of alteration within the breccia deposit. Finally, we compared breccia-derived material with outcropping shallow units and drill hole material from well K-36 (Fig. 14) to infer eruptive source lithologies and excavation depth. Collectively, field observations and drill cuttings information were used to reconstruct the pre-eruption setting (Fig. 15) and to infer an eruption scenario (Fig. 16). Additional information about the field study, together with an isopach map of the larger breccia deposit, is reported in the Online Resources.

### Pre-eruption volcaniclastic sequence

The pre-eruption stratigraphy outcropping around Víti crater consists mainly of 30‒40-m-thick hyaloclastite tuffs and pillow breccia, overlain by scoria fall or time-equivalent lava flows that is in turn covered by a breccia unit and a thin scoria fallout. The hyaloclastite sequence is intersected in the NE and SE sectors by a 2‒3-m-thick brecciated intrusion made of black, aphanitic basalt (Figs. 3, 4, 5, 6, 7, 13, and ESM 1).

The hyaloclastite tuff beds show a fine-grained, light-to-dark brown vitric matrix, embedding cm-to-dm-sized scoriaceous “flames” (individual and fragmented scoria welded and deformed; Fig. 4b). The pillow breccia beds include a range of randomly orientated angular clasts, from fine-grained up to block-sized, supported by a poorly sorted matrix. Large clasts are mostly non-vesicular to vesicular basalt, pillow fragments, and tephra. The matrix consists of fragmented and poorly sorted, coarse to small pebbles of altered glass and crystalline basalt, cemented by secondary minerals (typically calcite and zeolite) that partially fill the original pores (Fig. 4c). The recognized tuff and pillow breccia beds have thicknesses from 2 to 4 m, dipping ~ 5‒10° north-eastward (Figs. 3 and ESM 1). The contact between the tuff and breccia beds is irregular and typically erosive, as evidenced by scouring and truncation of underlying beds (Fig. 4b). In the northern sections, a scoria-dominated bed, indicative of a final magmatic phase, caps the top part of the hyaloclastite (Fig. 5a, b).

The hyaloclastite unit is overlain by scoria cone deposits (Figs. 3, 4, 5, and 6). In the northern sector, these scoria deposits lie over the hyaloclastite above a sharp erosive contact, with rare bomb sags (Fig. 5c). The main outcrop exhibits the cross-section of a remnant of a scoria cone ~ 4-m-thick in its central part (thinning out to ~ 0.5 m), including the following: (i) a ~ 0.05‒0.1-m basal fine-to-coarse ash and lapilli level, rich in altered lithics; (ii) a 1–2-m-thick spatter agglutinate with bombs; and (iii) ~ 1.5-m alternating massive, poorly vesicular, aphanitic lava flow and scoria fall deposits (Fig. 5a,c,d and Sec. 2‒4 in Fig. 13). Locally, the scoria fall are found alternating with dm-thick, laminated ash beds, likely representative of small ash flow deposits (Sec. 3 in Fig. 13). An ash-to-lapilli scoria level ~ 0.6-m thick characterizes the top of the scoria cone deposits, with an erosive sharp contact separating it from the overlying breccia deposit (Fig. 5e). The same hyaloclastite/scoria cone sequence can be observed within a collapsed block located just below the main outcrop (Figs. 3 and 5a). In all remaining sectors around Víti, the scoria cone deposits consist of non-vesicular (massive) to vesicular, aphanitic lava flows (Sec. 1 and 10‒13 in Fig. 13). To the northeast, an entire lava flow (~ 800 m-long) is observable, while in the other sectors, the lava bodies are buried below the breccia deposits, and only proximal portions are visible (Figs. 6, 11, and 12). These lava flows are locally fractured and, especially in the southeastern-southern sectors, are moderately to highly altered with abundant zeolite filling the original pore space. In the southeastern side, a dike that fed the lava flows is exposed cutting through the tuff and breccia beds (Figs. 6, 12 and Sec. 10 in Fig. 13).

An orange-to-brown, matrix- to clast-supported breccia, containing heterolithic, angular to subrounded lapilli to coarse blocks, hereafter “Hveragil breccia”, overlies the scoria cone deposits. This breccia is sourced from the Hveragil fissure and can be found surrounding the Víti crater outcrops, with a decreasing thickness (> 5 to < 0.5 m) to the northwest (Figs. 7, 9, 11, and 12). Though not analysed in detail, the componentry of this breccia appears to include mostly material derived from the hyaloclastite formations, with subsidiary silicic (felsite) clasts and crystalline intrusive basalts, as well as  fresh rhyolitic juveniles. On the north side, ash-rich tuff and rhyolitic tephra deposits are locally intercalated between the scoria cone/basaltic intrusion/hyaloclastite tuff and the Hveragil breccia (Figs. 7 and Sec. 4‒8 in Fig. 13).

Finally, a black, scoriaceous ash-to-lapilli fall deposit sits above the Hveragil breccia (Figs. 7, 8, 9, 10, 13). This scoria fall has a variable thickness (< 0.05‒1 m), but thickens in the northern side, indicating an eruptive source from a north or north-western vent or fissure. In almost all of the investigated sections, we find the Víti breccia deposits overlapping the scoria fall deposit typically with a gradual contact and locally with erosional surfaces where the scoria is missing (Sec. 13 in Fig. 13).

### Víti breccia stratigraphy

Five breccia deposits (1‒5, oldest to youngest) can be distinguished based on their components, grain size, and colour. The lack of exposure in some of the investigated areas due to intense alteration, or the presence of thick scree blanket, means that we cannot exclude larger thicknesses of some of the breccia deposits, or a larger extension of block- and juvenile-rich levels.

The outcropping breccias 1 to 4 show irregular shapes and variable thickness, with limited distribution around the NNE crater cluster. Appearance, grain size, texture, componentry, and thicknesses variation are shown in Figs. 7, 8, 9 and 13 (Sec. 3‒9) and can be described as follows:

- Breccia 1 consists of a lower dark grey, coarse ash dominated layer, and an upper orange breccia, clast-supported with subrounded to angular lapilli and minor blocks. The coarse ash layer is max. 1–2-cm thick and shows a bimodal grain-size distribution with peaks at − 3.5 and − 1 phi (8 and 2 mm; medium lapilli and very coarse ash), as well as an abundant tail of medium-fine ash (∼ 25 wt%). Components mostly include orange altered lava (~ 64%), pillow fragments (~ 21%), minor tuffs (~ 7%), old basaltic scoria coated with an alteration patina (~ 6%), and rare unaltered lava (~ 2%). The clast-supported layer is 5–35-cm thick, with a coarsely skewed grain size distribution, having a large mode in the lapilli fraction and a major tail of ash (∼ 35 wt%). Clasts comprise mainly old basaltic scoria (~ 49%) and altered lava (~ 38%), with minor altered tuffs (~ 5%), unaltered lavas (~ 4%), and black pillow fragments (~ 4%).
- Breccia 2 is a 10–80-cm-thick grey-greenish breccia, dominated by coarse-grained matrix supporting subrounded to angular lapilli and minor blocks. The matrix is coarsely skewed with a dominant peak at − 3 and a secondary one at − 4 phi (8 and 16 mm; medium lapilli), as well as a major tail of ash (∼ 35 wt%). Altered lava is the dominant component (~ 48%) and then brecciated clasts (~ 20%) and unaltered lava (~ 10%), with minor black pillow fragments (~ 7%), old basaltic scoria (~ 5%), altered tuffs (~ 4%), calcite fragments (~ 4%), altered green tuffs (~ 3%), and rare gypsum clasts (< 1%).
- Breccia 3 is an orange matrix-supported breccia, 10–100-cm thick, with subrounded to subangular lapilli and minor blocks. The matrix is coarsely skewed with dominant modes in the medium lapilli and a significant tail of ash (∼ 37 wt%). The components include mainly altered lava (~ 63%), subsidiary unaltered lava (~ 16%), minor altered tuffs (~ 6%), black pillow fragments (~ 6%), brecciated clasts (~ 5%), and old basaltic scoria (~ 4%).
- Breccia 4 is a greyish matrix- to clast-supported breccia 20–100-cm thick, with subrounded to angular lapilli and abundant blocks. The breccia matrix is coarsely skewed showing dominant modes in the medium lapilli and a large tail of fine ash (∼ 43 wt%). Components are dominated by altered lava (~ 76%), with minor altered tuffs (~ 8%), old basaltic scoria (~ 7%), unaltered lavas (~ 6%), black pillow fragments (~ 2%), and rare gypsum clasts (< 1%).

The breccia 5 has a ~ N-S elongate distribution with respect to the main Víti crater, with its thicker side towards the south (Figs. 2 and 3). Immediately north of the crater rim, the breccia is 1.5-m thick, thinning to ~ 1 m at 150-m distance, and reduced to zero within 300–400 m (Fig. 10). By contrast, the breccia is ~ 10-m thick immediately south of the crater, ~ 4-m thick at a 250-m distance, and is still ~ 1 m southward at the edge of erosive channels that likely removed part of the deposit (Figs. 3, 11 and Sec. 10‒14 in Fig. 13).

On the northern rim, breccia 5 is grey-greenish, matrix- to clast-supported breccia, block-rich with subrounded to subangular lapilli (Figs. 8, 9, and 10). The matrix is coarsely skewed and shows a large mode in the medium lapilli fraction and a large tail of ash (∼ 43 wt%; Fig. 8b). Altered lava fragments make up most of the fragments (~ 68%), with lesser unaltered lavas (~ 21%) and minor altered green tuffs (~ 4%), black pillow fragments (~ 3%), brecciated clasts (~ 2%), old basaltic scoria (~ 2%), as well as rare felsite and calcite fragments (< 1%; Fig. 8c,d). Surficial lapilli-sized material from the northern sector, just above the scoria cone deposits, is composed of unaltered (~ 21%) and altered lava fragments (~ 46%), with abundant old basaltic scoria (~ 18%), pillow fragments (~ 13%), and rare altered green tuffs (< 1%). In the northeastern sector, in contrast, unaltered (~ 33%) and altered lava fragments (~ 57%) predominate, with subsidiary pillow fragments (~ 5%), old basaltic scoria (~ 3%), and altered green tuffs (~ 2%; Fig. ESM 2).

On the southern rim, as on the northern, breccia 5 is grey-greenish in colour and has similar clast-type proportions, but shows a vertical stratification with diffuse contacts between three massive beds (Figs. 11 and 13). The basal bed consists of a fine-grained, matrix-supported breccia 0.5–1.5-m thick, with subrounded to angular lapilli and minor blocks (the latter more abundant in the upper bed). The matrix shows a large mode in the coarse ash to fine lapilli fractions and an abundant tail of medium-fine ash (∼ 33 wt%; Fig. 11b). The components are most exclusively unaltered (∼ 21%) and altered lava (~ 66%), with minor black pillow fragments (4%), brecciated clasts of grey tuff (∼ 4%), altered green and grey tuffs (~ 2%), as well as rare calcite (∼ 1%) and felsite fragments (∼ 1%; Fig. 11c). The middle bed is 1–4.5-m thick, matrix- to clast-supported, and contains a large fraction of angular to subrounded lapilli to blocks (up to 0.6-m size; Fig. 11a). The matrix grain size distribution is polymodal with peaks at − 0.5, − 1.5, − 2.5, and − 4 phi (1.4, 2.8, 5.6, and 16 mm) and a very large tail of ash (∼ 50 wt%; Fig. 11b). The matrix componentry reveals lesser amounts of unaltered (∼ 5%) and altered lava (~ 44%) compared to the lower bed and include a significant amount of ash-lapilli-sized, moderately to highly vesicular rhyolitic pumice (~ 16%) and fresh basaltic scoria (~ 2%), with abundant altered pillow fragments (~ 13%) and green altered tuffs exhibiting mineralized circular vesicles (~ 10%), minor old basaltic scoria (~ 7%), and rare (< 4%) felsite, brecciated green tuffs, and calcite fragments. A few pumice clasts contain small mafic bands or exhibit mingled textures between scoria- and pumice-looking material (Fig. 11c). Though not quantified, felsic, and gabbroic clasts are more abundant in the fine block-sized fraction. The upper bed is < 0.5–3.5-m thick; its matrix is coarsely skewed with a large mode in the medium lapilli fraction and a strong peak at − 4 phi and has a lower ash content (< 40 wt%) than the lower or middle bed (Fig. 11b). As in the lower bed, lavas (∼ 9% unaltered and ~ 73% altered) are the dominant components, but a significant amount of altered brown-grey tuff (∼ 9%) is present, together with rare fragments of altered pillow, green altered tuffs, old basaltic scoria, calcite fragments, and felsite (< 6% in total). Brecciated clasts are absent, while fresh rhyolitic pumice is a rare matrix component (~ 2%; Fig. 11c). Surficial breccia sampled in the proximal areas of the southeastern and southern ballistic fields shows a similar componentry to the northern side and is dominated by unaltered (21‒45%) and altered lava fragments (38‒57%), with abundant old basaltic scoria (2‒18%) and pillow fragments (3‒13%). Yet, more abundant green altered tuffs (< 10%), felsite (< 6%), and rare altered grey tuffs (< 3%) can be found (Fig. ESM 2).

### Componentry and distribution of blocks

The upper portion of Breccia 5 is rich in coarse blocks (> 25-cm size) that are found predominantly in large ballistic fields in the northern, eastern, southeastern, and southern sectors of Víti crater (Fig. 2). In the northern, eastern, and southeastern sectors, dense ballistic fields cover the breccia deposit over a distance of ~ 500, ~ 150, and ~ 200 m, respectively, whereas on the southern side, only a few scattered clusters of ballistics are found < 100 m from the crater rim (Fig. 2). Ballistic blocks are mostly preserved either above or partially immersed in the breccia without obvious impact craters (Fig. ESM 3), suggesting that the ballistics fell onto the breccia during its emplacement.

Coarse ballistic blocks are mostly of four lithological types (Fig. ESM 3): lava clasts composed of round to angular, non-vesicular to vesicular, aphanitic basaltic lava which varies from unaltered (49%) to variably altered (27%); hyaloclastite tuffs made of glassy matrix embedding deformed scoria, unaltered (16%) to variably altered (7%); hyaloclastite pillow breccia (1%) containing cm-sized pillow rinds and core fragments of varying textures incorporated in a fine- to medium-grained matrix (see also Fig. 4c); and silicic felsite (2%). Rare small blocks (< 25 cm) also include brecciated clasts made of altered and fragmented greenish tuffs or massive lava embedded in an alteration mineral matrix.

Lava clasts, hyaloclastite tuffs, and pillow breccia are found in fresh exposures of the Víti crater wall on both north and southern sides (Figs. 3, 4, 5, and 6). Block types are differently distributed around the crater and generally mirror the outcropping lithologies (Figs. 2 and ESM 3). Unaltered lavas are uniformly dispersed over the northern and eastern ballistic impact area, while altered lavas are dominant on the southeastern and southern side. Hyaloclastite tuff blocks are prevalent on the northern side, found in minor amounts on the eastern side, but are rare in the southern sector. Clasts of hyaloclastite pillow breccia and felsite are less common and mostly found in the northern and eastern fields, while rare, brecciated clasts occur in the NE-E side.

### Insight into shallow geology from drill hole K-36

Over the past 40 years, the large geothermal system in Krafla (∼ 40 km^2^) has gradually been explored and developed, and currently a 60 MW geothermal power station is in operation. By 2020, a total of 43 exploration and production wells, with depths from 985 to 2894 m, had been drilled in the Krafla geothermal system. At least three times magmatic bodies (and their respective contact aureoles) have been encountered (Hólmgeirsson et al. 2010). Superheated steam at the bottom of wells IDDP-1, K-39, K-36, K-25, K-10, and K-4 indicates “close-to-magma” conditions, with K-39 and IDDP-1 returning glass in cuttings (Hólmgeirsson et al. 2010). These wells were all drilled near the Víti area, as was the ~ NW-directed well K-36, which was drilled for a length of 2500 m on a tangent to the NE side of the crater (Fig. 2). The stratigraphy penetrated by this well resembles that seen in well IDDP-1 and K-25 (Gautason et al. 2007; Guðmundsson et al. 2007, 2008a,b; Schiffman et al. 2014). The upper 1152 m of well K-36 consists mostly of basaltic lavas and hyaloclastite formations, reflecting changing climatic conditions through time as the volcanic pile accumulated. The overall stratigraphy reconstructed from drill cuttings (mm-sized grains) and, shown in Fig. ESM 4, can be divided into five main sequences:

- 14–100 m: Oxidized scoria levels in the shallow (< 20 m) ground and basaltic tuffs overlie a series of Holocene lavas intercalated with hyaloclastite consisting of mixtures of aphanitic basalt breccias and tuff. The basaltic lavas are blackish to dark-grey, fine-grained, and aphyric. Some lavas are fractured, and the veins are filled with chalcedony. Hyaloclastites are mostly light-to-dark grey in colour and consist in highly mixed samples of basalt breccia, tuff, and basalt lumps (Fig. 14a). Rock alteration intensifies with depths with an increasing abundance of zeolites, calcite, pyrite, clay, and subordinate quartz. Minor amounts of clay and pyrite coat the vesicles in the uppermost lavas and tuffs.
- 100–234 m: Consolidated and greenish altered basaltic hyaloclastite intercalated with lavas and minor breccias. The hyaloclastite consists of fine- and medium-grained tuff with clasts having small spherical vesicles; vesicles and groundmass are filled or replaced by alteration minerals, primarily calcite and pyrite (Fig. 14b). The lavas occur as several thin layers that are primarily fine- to coarse-grained (crystals < 0.05 to > 2 mm) and aphyric, though some contain phenocrysts of plagioclase or altered olivine. The basaltic breccia includes a fine-grained mixture of clasts of light brown basalt and tuff, strongly cemented together. Alteration minerals include chalcedony, calcite, zeolites, pyrite, and subordinate quartz (Mortensen et al. 2015).
- 234–822 m: Thick sequence dominated by moderately altered basaltic lavas; predominantly medium- to coarse-grained (crystals 1 to > 2 mm) basalt with intercalated aphanitic basalt, green altered tuff, and basaltic breccia. About half of the sequence of basaltic lavas contains plagioclase phenocrysts (Fig. 14c). Between 476 and 498 m, there is light green moderately altered hyaloclastite consisting of a basaltic tuff with clasts that are fine- to medium-grained and aphyric. Rock alteration in the upper sequence involves clay minerals with abundant calcite, and pyrite and subordinate zeolite, with chlorite appearing at 286 m depth, and epidote at 714 m (Mortensen et al. 2015).
- 822–1008 m: Light grey to light green, highly altered, hyaloclastite is mostly made of fine- to medium-grained, aphyric basaltic tuff. Hydrothermal alteration has made the rock light green, with abundant calcite in vesicles and groundmass. Within the hyaloclastite are a few thin coherent units of altered, fine- to coarse-grained basalt, and basaltic breccia.
- 1008–1152 m: Basalt and basaltic andesitic lava sequence. The upper part of the sequence is represented by light-green cryptocrystalline basaltic andesite to andesite with very small plagioclase laths in the groundmass. Below 1040-m depth, the sequence consists of homogeneous and predominantly aphyric, greenish-grey basaltic lava flows alternating from fine- to coarse-grained in granularity, and thin interlayers of basaltic breccia with vesicular clasts. White felsite fragments were recovered in the drilling interval 1112‒1116 m, 1120‒1122 m, and 1146–1152 m, with no clear evidence of intrusive contact with the host basalt (Fig. 14d). Single felsite veins or multiple veining features may be the source of these fragments. Alteration is extensive and minerals include wollastonite (> 270 °C), quartz, epidote, and chlorite, as well as pyrite and calcite (Mortensen et al. 2015).

Below 1152 m the well enters a dike complex that is not included in our descriptions because no cuttings were retrieved below 1600 m.  However, petrophysical logs (neutron-neutron intensity, gamma and resistivity logs) indicate that below 1600 m, stratigraphy entails lava sequences, basaltic intrusions of variable thickness (5‒10 m to 200 m), and scarce thin felsic dikes.

## Discussion

### Pre-eruptive setting

Detailed analyses of the very shallow geology outcropping in the Víti crater, combined with the drill cuttings data from well K-36, give insight into local pre-eruptive setting, and provide stratigraphic and lithological controls pivotal for understanding the dynamics of the eruption (Mastin 1991; Gallagher et al. 2020).

The subsurface geology around the Víti craters is dominated by hyaloclastite deposits formed during glacial periods, and basaltic lavas during postglacial and interglacial times, as well as (localized) tephra units from nearby volcanic systems (Sæmundsson 1991). Surficial lava flows appear to have been fed from the Mt. Krafla by fissures, as by scattered scoria cones and fissures northwest and west of Víti crater (Figs. 1 and 2). Our mapping shows that, before the Víti eruption, a scoria cone complex overlay post-glacial eroded hyaloclastite deposits. The cone edifice was likely bigger than the observed remnants (max. 4-m thick) and possibly had a larger size of the cone on the ~ N, which has proximal deposits ~ 10-m thick and smaller lava flows (Figs. 2, 15). This scoria cone complex had one or more vents, as indicated by the types and distribution of the deposits. Intercalation of scoria beds with welded fallout beds and/or lava flows found in the north of Víti crater indicate changes in eruption style from Strombolian to Hawaiian and vice versa during the course of the eruption that formed the scoria cone (Parfitt and Wilson 1995; Martin and Németh 2006). Moreover, the presence of pyroclastic ash flow beds might suggest some interaction between magma and water during the eruptive phases (Houghton et al. 1999). Whereas explosive activity appears to have dominated the northern portion, thick and large lava flows were emitted in the northeast and southward sectors (Fig. 15). The scoria cone complex deposits were later covered by the breccia erupted from the 9000-year-old Hveragil eruption (Sæmundsson 1991; Jónasson 1994). This suggests that the scoria cone formed soon after the last glacial period (~ 10,000 years ago), which is consistent with the formation of other postglacial craters and fissures formed as result of a pressure drop following glacial retreat/melting (Weisenberger et al. 2015).

The moderate to strong alteration of tuffs, pillow breccia, and lava fragments in the northern breccia deposits (1‒4), as well as in the breccia ejected SE-ward and S-ward, is consistent with long-term exposure of the bedrock to hydrothermal fluids (thousands of years) prior to the eruptive event of 1724. The lithologies presents as clasts in the breccia derived from altered hyaloclastites in the northernmost area (Fig. 7) and from the fractured and altered lava deposits in the southeast and southern areas (Figs. 12 and 13). Geothermal features such as hot spring conduits and mud pools are most commonly found close or above faults and fracture systems (Curewitz and Karson 1997; Germa et al. 2013; Báez et al. 2017; D’Elia et al. 2020). At Víti, an NNE-oriented fault has been detected at depth in several wells underneath the main crater (Weisenberger et al. 2015). Moreover, the intensely fractured and altered bedrock NW and SE of the main crater might be associated with a NW-directed fault not previously reported (Fig. 15). This would represent the continuation of one of the faults east of Hveragil reactivated during the Krafla Fires (Ármannsson et al. 1987). Collectively, these all suggest a structurally controlled rise of magma that fed the inferred scoria cone, as well as that of magmatic and hydrothermal fluids altering the rocks prior to the Víti eruption (Rowland and Simmons 2012; López-Rojas and Carrasco-Núñez 2015; Kennedy et al. 2018; Scudero et al. 2019).

Finally, a lobe or multiple lobes of the Hveragil breccia were unevenly distributed over the scoria cone deposits during the 9000-year-old eruption, followed by a scoria fallout erupted by a nearby vent/fissure.

### Source lithologies of Víti breccia

The lithological nature and distribution of clasts found within the Víti breccia deposits indicate a wide range of geological source types and depth involved during different phases of the eruption, similarly to other known steam-driven and phreatomagmatic explosive events (Mastin 1991; Geshi et al. 2011; Breard et al. 2014; Pittari et al. 2016; Graettinger and Valentine 2017; Gallagher et al. 2020; Montanaro et al. 2020). Here, we were generally able to fingerprint the specific host rocks involved at different phases of the eruption by correlating lithologies in the ejecta with drill cuttings material (Mastin 1991; Gallagher et al. 2020).

The lapilli and block fractions of breccia deposits 1‒4 are mostly altered lava clasts, with minor older basaltic scoria (coated with alteration patina), hyaloclastite black pillow and tuff fragments, and minor unaltered lava (Fig. 8). Such components represent the very shallow rocks (< 60 m) now exposed in the crater walls of the NNE crater cluster and around the main crater, with altered material mostly sourced from the intensely altered northernmost sector (Fig. 7).

In the southern sector, the lapilli-sized fraction in the lower bed of Breccia 5 is rich in unaltered and altered lava fragments, with lesser unaltered aphyric lava, pillow and brecciated tuffs, with minor black, old basaltic scoria, altered grey tuffs, and calcite fragments. Comparison of this componentry with that from well K-36, and especially the presence of altered/brecciated grey tuff and calcite, suggests that erupted lithologies derive predominantly from the hyaloclastite and lava sequence found in the upper ~ 200 m (Figs. 14 and ESM 4). Fractured hyaloclastite with calcite-bearing veins are also commonly found in the shallow portion of Vesturhlíðar field (Ármannsson et al. 2007).

The lapilli-to-block-sized fraction of the middle bed includes a lesser amount of unaltered and altered lava fragments compared to the lower bed, but more abundant clasts of altered green tuffs with pyroclasts having mineralized circular vesicles, and older basaltic scoria. Additionally, there is large input of freshly fragmented, moderately to highly vesicular rhyolitic juvenile with minor fresh basaltic scoria and rare felsite and gabbroic clasts (especially in the fine block sizes). Hyaloclastite and lava from shallow levels (< 200 m) remain a large source during this eruptive phase. A small but significant contribution however comes from deeper levels as indicated by (i) altered green tuffs typically found at 200‒400-m depth and (ii) felsite and gabbroic clasts typically sitting at > 1100-m depth (Figs. 14 and ESM 4). The presence of freshly fragmented magma with felsite and gabbroic clasts suggest that the rhyolite usually found at ~ 2-km depth intruded into these shallower units prior to the eruption.

Lapilli-to-block-sized clasts of the upper bed are dominated by unaltered to altered lava, with minor pillow breccia, unaltered brown-orange tuffs (especially in the block size), altered grey tuffs, old basaltic scoria and rare brecciated tuffs, felsite, calcite fragments, and rhyolite pumice. This suggests that explosions during the final phase of the eruption were disrupting and/or recycling mostly hyaloclastite and lava from shallow levels, whereas input from deeply seated lithologies and magma was negligible.

The breccia 5 in the northern sector does not show different beds, yet componentry is similar to that of lower and upper bed from the southern sector, with dominant altered lavas and subsidiary/minor pillow fragments, old basaltic scoria, and brecciated/altered tuffs. Small amounts of felsite and very rare gabbroic clasts and fresh rhyolite pumice indicate that the material erupted northward was mostly sourced from < 200-m depth, excavating shallow lithologies during the initial and final phases of Víti crater formation. The large abundance in old scoria lapilli and bombs in the very surficial breccia (especially from the section above the scoria cone remnant) might be explained by a northward enlargement of the crater during the final phase.

Despite the correlation existing between breccia matrix and drill cutting components, caution must be exercised in the source lithology interpretation due to (i) subsurface mixing and sorting occurring during the arrival of deep-seated material (Graettinger et al. 2015a) and (ii) the efficiency of steam- or magmatic-driven fragmentation of host rock into ash-fine lapilli sizes (Mayer et al. 2015; Montanaro et al. 2016b). For example, the matrix of breccia 5 lacks felsite and gabbroic material, although this is more abundant in the fine-to-coarse blocks. This might indicate that fragmentation processes were less efficient for the deep-seated lithologies, or less recycling abrasion of deep-seated clasts (Kueppers et al. 2006; Campbell et al. 2013).

Finally, we observed a large number of fragments from the inferred scoria cone within the breccia 5. To assess the volume of the inferred cone, we compared the volume of breccia 5 with that of the excavated bedrock. The measured thicknesses of breccia 5 were used to produce an isopach map and a digital model (Fig. ESM 5). Employing the isopachs, we calculated a volume of ⁓ 5 × 10^5^ m^3^ for the breccia 5. We then used the 2-m resolution ArcticDEM digital terrain (Porter et al. 2018) to estimate the approximate volume between the lake level (~ 622 m a.s.l.) and the base of the scoria cone deposits (see red line in Fig. ESM 5), i.e., the minimum volume of excavated hyaloclastite, which is equal to ⁓ 4 × 10^5^ m^3^. Considering that we do not know (i) the depth of the lake and (ii) the precise thickness distribution of the breccia 5, as well as that (iii) part of the southern portion of the breccia has been eroded, we are likely underestimating both volumes. However, our estimation suggests a minimum scoria cone complex volume in the order of ⁓ 1 × 10^5^ m^3^, corresponding to ⁓ 20% of breccia 5. This estimate is consistent with the hypothesis of the existence of a large scoria cone complex prior to the eruption.

### Eruption trigger and mechanism

Potential triggers of the explosive activity at Víti and eruptive scenarios can be inferred from several lines of evidence, including subsurface stratigraphy and tectonic structures, as well as the alteration state and stratigraphy of the Víti breccia deposits.

Schlieren of quenched basalt found within rhyolitic pumices of the middle bed of Breccia 5 are typical flow structures related to the development of mingling and mixing dynamics between two magmas (Perugini and Poli 2012). These glass textures provide proof of interaction between the magmas and suggest that the injection of basalt likely perturbed the chemical and thermal equilibrium of the rhyolite melt (Spera and Bohrson 2018). Basaltic injections can mobilize silicic magmas triggering an eruption, as occurred at Askja in 1875 (Sigurdsson and Sparks 1981), or at Hveragil in Krafla (Jónasson 1994), as well as during the 2010 Eyjafjallajökull eruption (Sigmarsson et al. 2011). Previous experimental and numerical studies (Perugini et al. 2010; Montagna et al. 2015; Semenov and Polyansky 2017) suggest that the type of glass textures observed in mingled juveniles at Víti could reflect timescales of mingling of mafic and silicic melts on the order of hours to days before an eruption. This hypothesis was confirmed by a petrological study of Rooyakkers et al. (2021), who found that crystals in the Víti’s rhyolite pumices do not show evidence of late-stage heating or re-equilibration with more mafic melt, thus implying a mixing-mingling time scale of at most several hours.

Rhyolitic melts have been previously found encased within basaltic dikes at ~ 2-km depth in wells IDDP-1 and K-39, located in the Vítismór and Leirbotnar geothermal fields, and found at the crossing or in proximity of faults (Fig. 1; Elders et al. 2011; Mortensen et al. 2015). However, intrusions of deeper magma prior to, and during, the Krafla Fires or many earlier basaltic eruptions, did not disturb shallow-seated rhyolitic magma bodies. The reason may reside in the geological setting prior to the basaltic intrusion. Víti crater lies above a N-S oriented fault system and within Vesturhlíðar, one of the highest temperature geothermal fields in Krafla (Fig. 1; Weisenberger et al. 2015 and references therein). The new field evidence indicates the presence of intensely fractured and altered zones likely associated with a NW-directed fault. Taken together, it appears that an approximately N-S–NW–SE cross fracture zone existed at the site of Víti before the eruption (Fig. 16). This is consistent with the fact that highly fractured zones can act as preferential pathways for magma and magmatic-hydrothermal fluids in time (Tentler 2005; Rowland and Simmons 2012; Gonnermann and Taisne 2015; Maccaferri et al. 2019). We further observed that in most of the altered clasts from the scoria cone deposits, as well as in ejected brecciated tuffs from shallow levels, secondary minerals fill original pore space and fractured veins (Figs. 14 and ESM 3). These findings suggest that alteration likely reduced porosity and permeability of portions of the scoria cone, and of shallow parts of the fractured hydrothermal system via mineral sealing (Browne and Lawless 2001; Rosi et al. 2018; Prause et al. 2020). Such a setting could have promoted local overpressures high enough to lead to explosive destabilization (Mayer et al. 2015, 2016; Scolamacchia and Cronin 2016; Heap et al. 2019).

Breccias 1 to 4 were erupted in the northern sector by explosions producing a ~ NNE elongate crater cluster. They include mostly unaltered and altered clasts from the shallow lava, hyaloclastite, scoria fallout, and aphyric basalt units, and no juvenile pyroclasts. This might suggest that the input of mass and energy (> *T*) deriving from the intrusion could have rapidly heated and pressurized the pre-existing convecting hydrothermal fluids in fractures and aquifers within faults at the site of Víti. Heat and gas cause rapid propagation of cracks leading to boiling within fractures and pores (Germanovich and Lowell 1995). Extensional stresses and overpressures built up and eventually caused the country rock to fail, initiating decompression and an explosive eruption. Different pressurisation pathways and different yield strengths of country rock can control crater-forming processes (Lube et al. 2014; Macorps et al. 2016; Montanaro et al. 2020). Thus, the presence of a thicker and stronger sequence of spatter and lava lithologies, forming the bulk of the inferred scoria cone complex, initially contained the overpressure produced by boiling fluids. Conversely, the altered hyaloclastite and reduced thickness of the scoria cone deposits in the northern sector might explain why the eruption began in this area (Fig. 16).

The continuous pulse of heat and gas from the magmatic intrusion further pressurized hydrothermal fluids until the tensile strength of the scoria cone “cap” was overcome (Browne and Lawless 2001; Rosi et al. 2018; Stix and Moor 2018; Heap et al. 2019; Gallagher et al. 2020; Montanaro et al. 2020). The breaking of relatively hard, low porosity spatter and lava likely led to an initial bottom-up explosion, eventually breaking to the surface with low energy and emplacing a small breccia deposit (lower bed; Fig. 16). The breaching of levels capping a pressurized hydrothermal system, such as the scoria cone, then led to a top-down rarefaction wave followed by expansion of flashing fluids that fragmented and ejected clasts (Germanovich and Lowell 1995; Kilgour et al. 2010; Gallagher et al. 2020; Montanaro et al. 2020). This eruption phase produced a wider ejection of larger particles and a thicker breccia dominated by hyaloclastite and lavas from shallow-intermediate depths (middle bed). The significant amount of fresh rhyolite within the lower half of the middle breccia (Fig. 11), together with the presence of felsite and gabbroic material, suggests that while the system breached at the surface, magma was fragmenting within the basaltic dike complex at ~ 1200-m depth. The highly vesicular rhyolitic pumice may indicate a lack of magma-water interaction during the fragmentation process (White and Valentine 2016), such that decompression and gas exsolution of the rising rhyolitic magma might have driven the fragmentation at depth, in concert with host rock disruption by the flashing of rapidly heated fluids (Fig. 16). Such deep-rooted explosions resulted in churning and mixing of fragmented magma and rock debris that were incrementally transported upward to optimal depths (less than about ~ 200–250 m; Ross and White 2006; Valentine et al. 2014; Sweeney and Valentine 2015), and eventually erupted. A deepening of the fragmentation level, or decrease in gas exsolution, may have resulted in increasingly confined “non-erupting” explosions, thus reducing the contribution of juvenile and deep-seated material (Sweeney and Valentine 2015; Cassidy et al. 2018), as observed in the middle bed of the breccia. Continuous flashing of fluids likely fuelled shallow explosions and subsequently enlarged the crater (Valentine et al. 2015; Gallagher et al. 2020; Kennedy et al. 2020; Montanaro et al. 2020). During this final phase, mostly shallow hyaloclastite and recycled fallback material was erupted to produce a thick massive breccia (upper bed; Fig. 16). Collapses also carried large blocks into the northern side of the crater (Figs. 3, 4, and 16).

The dominant shallow explosion depths throughout the eruption, as well as the texture and componentry of the breccia deposits, imply deposition from ballistic curtains, with extremely rapid sedimentation from a spreading and collapsing jet (Fig. 16; Graettinger et al. 2015b; Graettinger and Valentine 2017). The breccia distribution pattern also indicates that strongly asymmetrical southward blasts deposited the thickest breccia beds, with a few sporadic north, east, and south eastward jets producing thinner breccia deposits and ballistic fields (Breard et al. 2014; Graettinger et al. 2015b). The lava lithologies dominating in the north and northeastern ballistic fields suggest that the majority of these blocks was erupted during the disruption of the main scoria cone, whereas jets carrying clasts of tuff, pillow breccia, and felsite were erupted later during later excavation stages (Breard et al. 2014).

## Conclusion

A new stratigraphic reconstruction of the breccia deposits erupted from Krafla’s Víti crater, along with characterization of the lithologies involved in the explosions through analyses of surficial outcrops and drill cuttings, has enabled the fingerprinting of the eruption trigger, source depths, and the mechanism of each eruptive phase contributing to the crater’s formation.

Injection of a mingled rhyolite beneath a pre-existing convecting hydrothermal system, causing a heat pulse, likely triggered the Víti eruption. Stratigraphic boundaries, along with variable degrees of alteration affecting porosity, permeability, and likely host rock strength, played a key role in eruption dynamics. Heat and gas pressure from magma below was focused into pre-existing NNE oriented faults and fractures, to overpressure shallow hydrothermal fluids until the scoria cone “cap” was disrupted and rapid fluid decompression began. The presence of a pre-existing altered and weak zone led to the first narrow/localised series of explosions, initially breaking and ejecting only shallow sub-surficial material. The second phase was laterally much more extensive and, overall, more violent with wider ejection of larger particles and formation of thicker deposits. This phase began when pressurized fluids broke through a relatively stronger scoria cone “cap”, and shallow weak and porous lithologies were disrupted and ejected from the shallow hydrothermal reservoir. Concomitantly, juvenile material and deep-seated lithologies were transported upwards and increasingly into an “eruptible depth range” by deep-rooted explosions. The final phase was dominated by flashing of shallow fluids and crater enlargement with occasional collapses.

Our results suggest that the Víti eruption can be classified as a magmatic-hydrothermal type (Browne and Lawless 2001; Rooyakkers et al. 2021) and was the result of a complex interaction between a rhyolite intrusion and the pre-existing convecting hydrothermal system existing in Vesturhlíðar. Moreover, our findings indicate that a shallow and thin hyaloclastite sequence hosting hot geothermal fluids (e.g. Vesturhlíðar field) and capped by low porosity and permeability lithologies (e.g. altered scoria cone complex and/or massive, thick lava flows) can be more susceptible to explosive failure in case of shallow magmatic intrusion(s). In contrast, intrusions occurring below thicker hyaloclastite sequences that host colder geothermal fluids (e.g. Leirbotnar and Vítismór), and lack of shallow cap level, may result solely in intense fracturing and degassing at the surface.

In a broader sense, our findings suggest that in active hydrothermal environments, the assessment of potential eruptive scenarios hinges on a detailed understanding of local geology, extent of hydrothermal systems, and on the local tectonic setting. Availability of detailed subsurface information, as is the case for Krafla, can help significantly in constraining estimates of the number and nature of possible triggers and eruptive mechanisms.

## Supplementary Information

Below is the link to the electronic supplementary material.

Supplementary file1 (DOCX 13340 kb)
