# Supplementary material for: Stratigraphic reconstruction of the Víti breccia at Krafla volcano (Iceland): insights into pre-eruptive conditions priming explosive eruptions in geothermal areas
Source: Bull Volcanol. 2021 Nov 2;83(11):81. doi: 10.1007/s00445-021-01502-y (PMC8563691; doi:10.1007/s00445-021-01502-y)
Supplement: Supplementary file 1 — Supplementary file1 (DOCX 13340 kb) [file 445_2021_1502_MOESM1_ESM.docx]

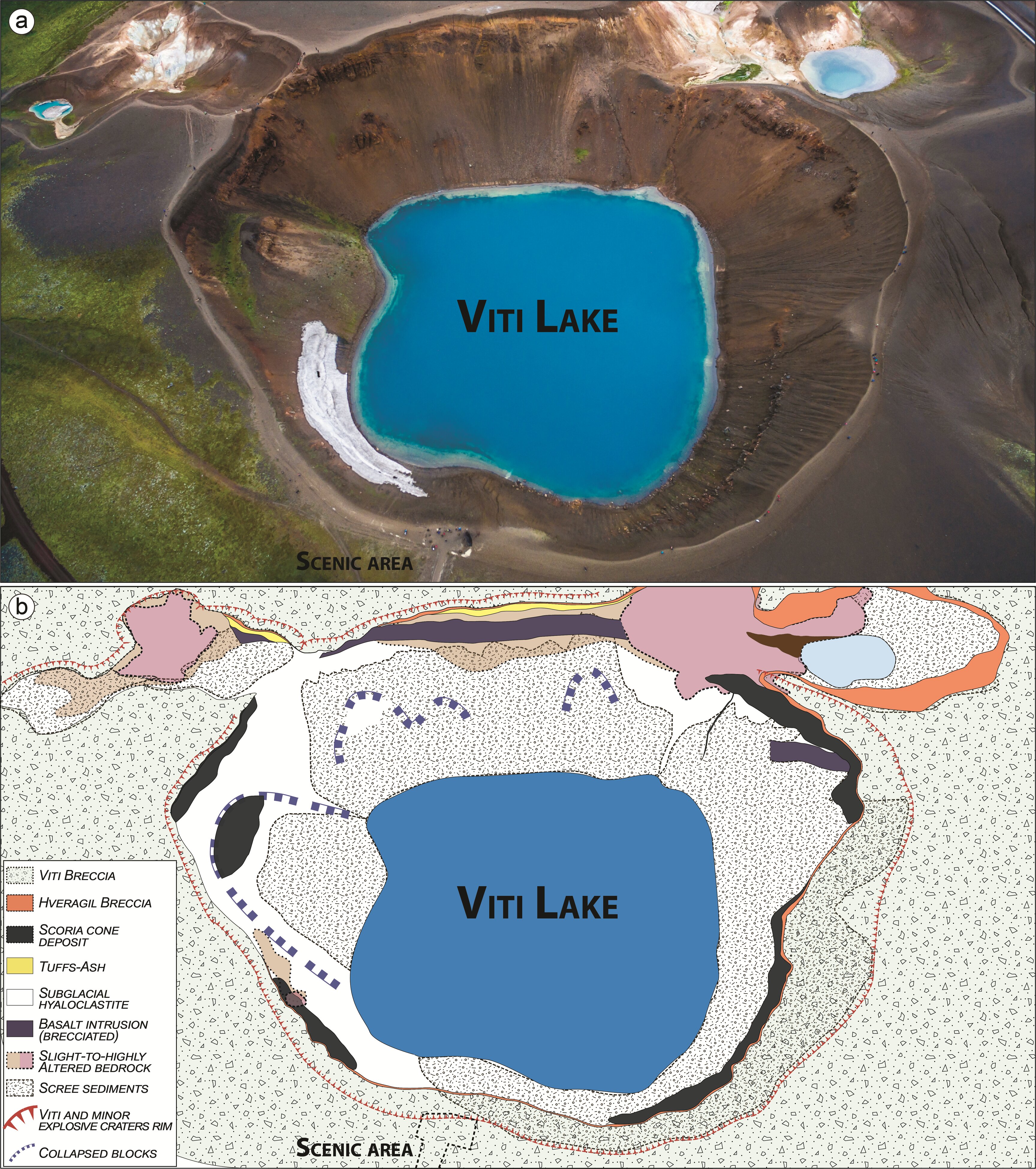


**Fig. ESM 1:** Aerial view (**a**) and geological map (**b**) of the Viti crater, and surrounding areas showing the main outcropping breccia, scoria cone, lava flow, hyaloclastite and basalt intrusion units covered by scree sediments. The Víti crater aerial photo in **a** is reproduced by kind permission of the photographer, Enrico Pescantini ([www.pescart.com](http://www.pescart.com)).


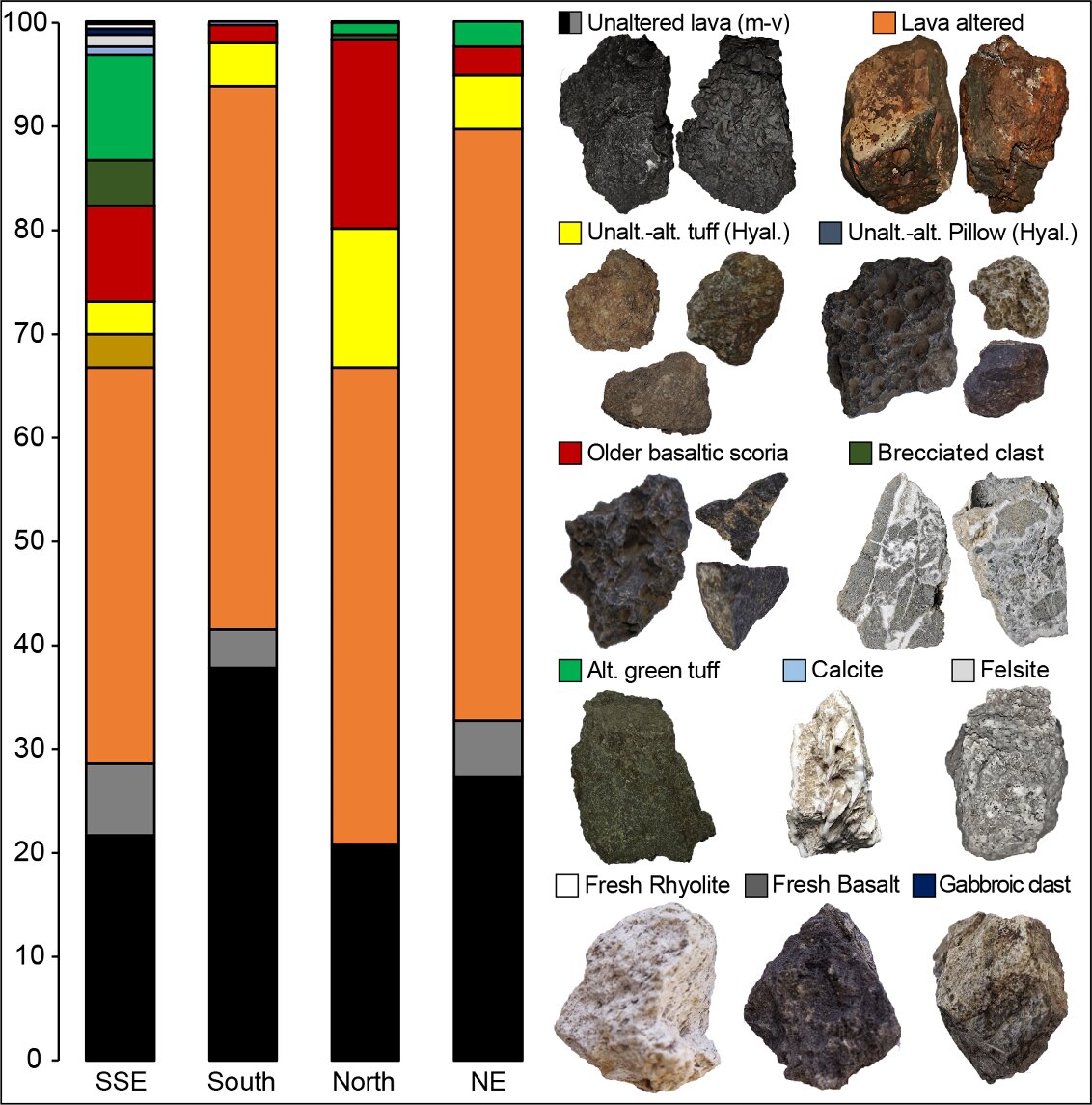


**Fig. ESM 2:** Componentry of surficial material of breccia 5 from the main ballistic fields area, determined from point counts (min.300 grains) of 1‒8 mm fraction in vol.%, and main components specimens.

**
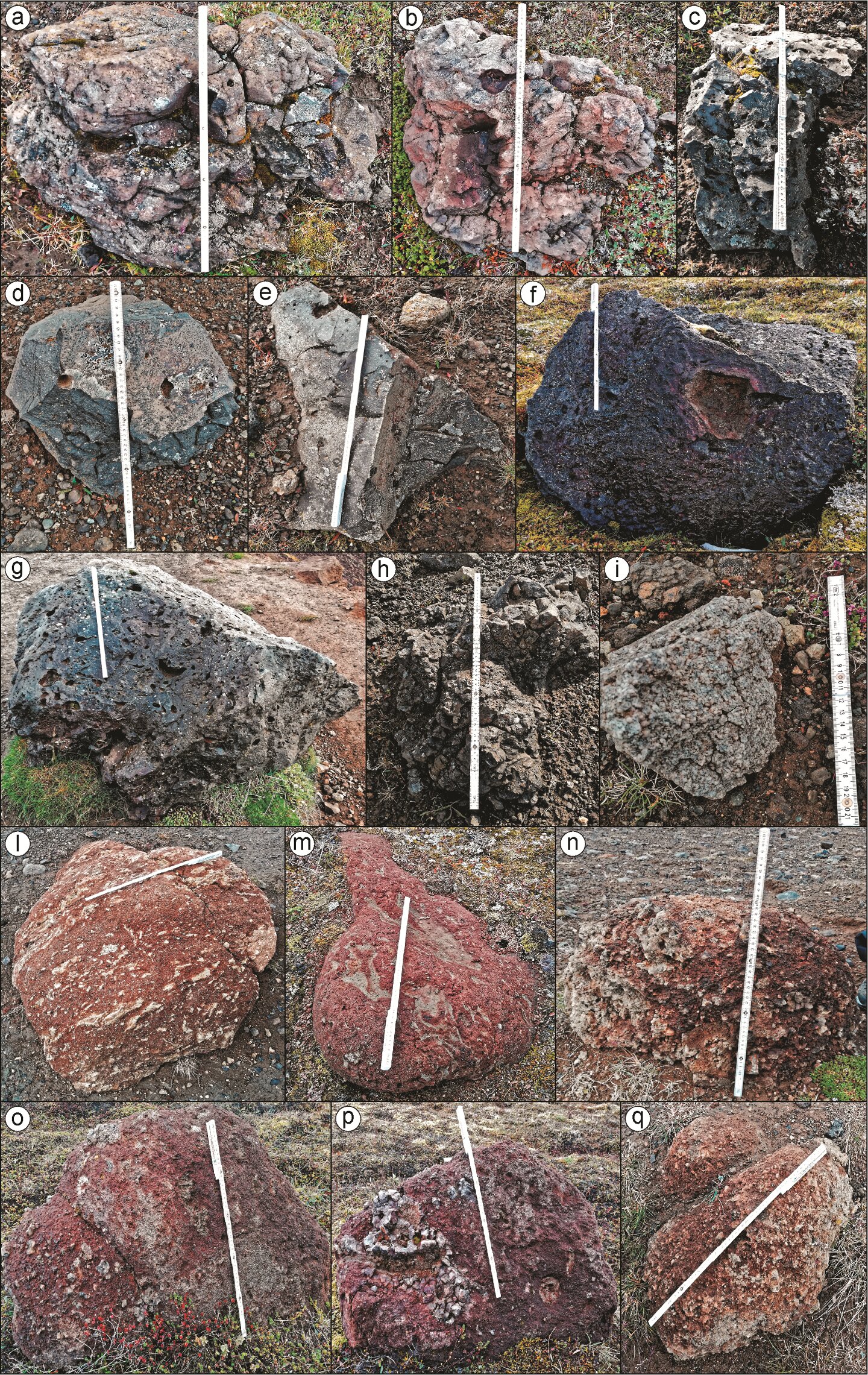
**

**Fig. ESM 3a:** Main lithologies of the coarse ballistic blocks (>25 cm) from the northern sector: **a‒h** unaltered to variably altered non-vesicular to vesicular lava; **i** felsite; **l‒m** hyaloclastite tuffs; **n‒q** hyaloclastite pillow breccia. The ruler used as scale in the pictures is 50 cm long.

**
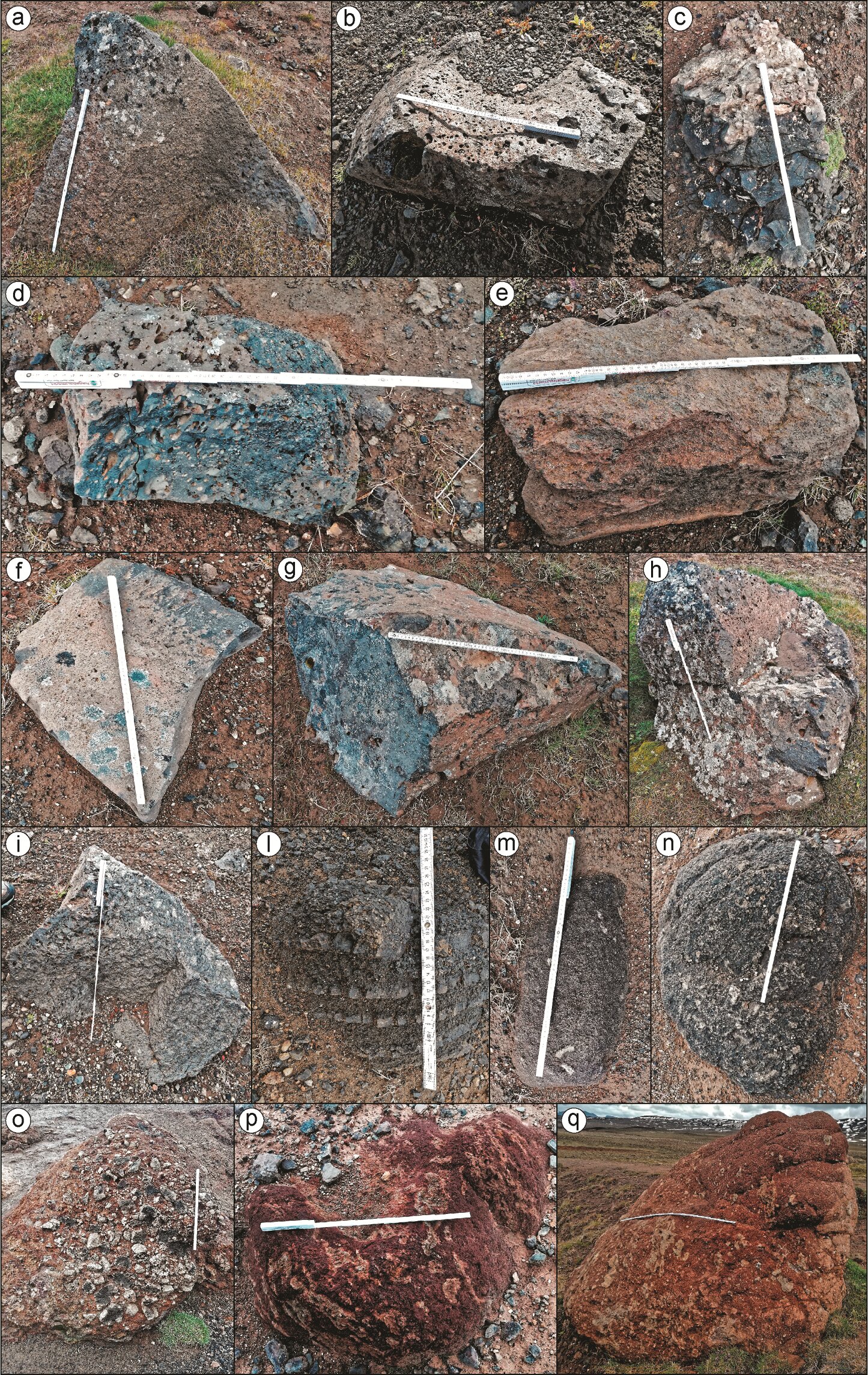
**

**Fig. ESM 3b:** Main lithologies of the coarse ballistic blocks (>25 cm) from the NNE-E sectors: **a‒i** unaltered to variably altered non-vesicular to vesicular lava; **l** alternating lava and scoria lapilli; **m‒o** hyaloclastite pillow breccia; **p‒q** hyaloclastite tuffs. The ruler used as scale in the pictures is 50 cm long.


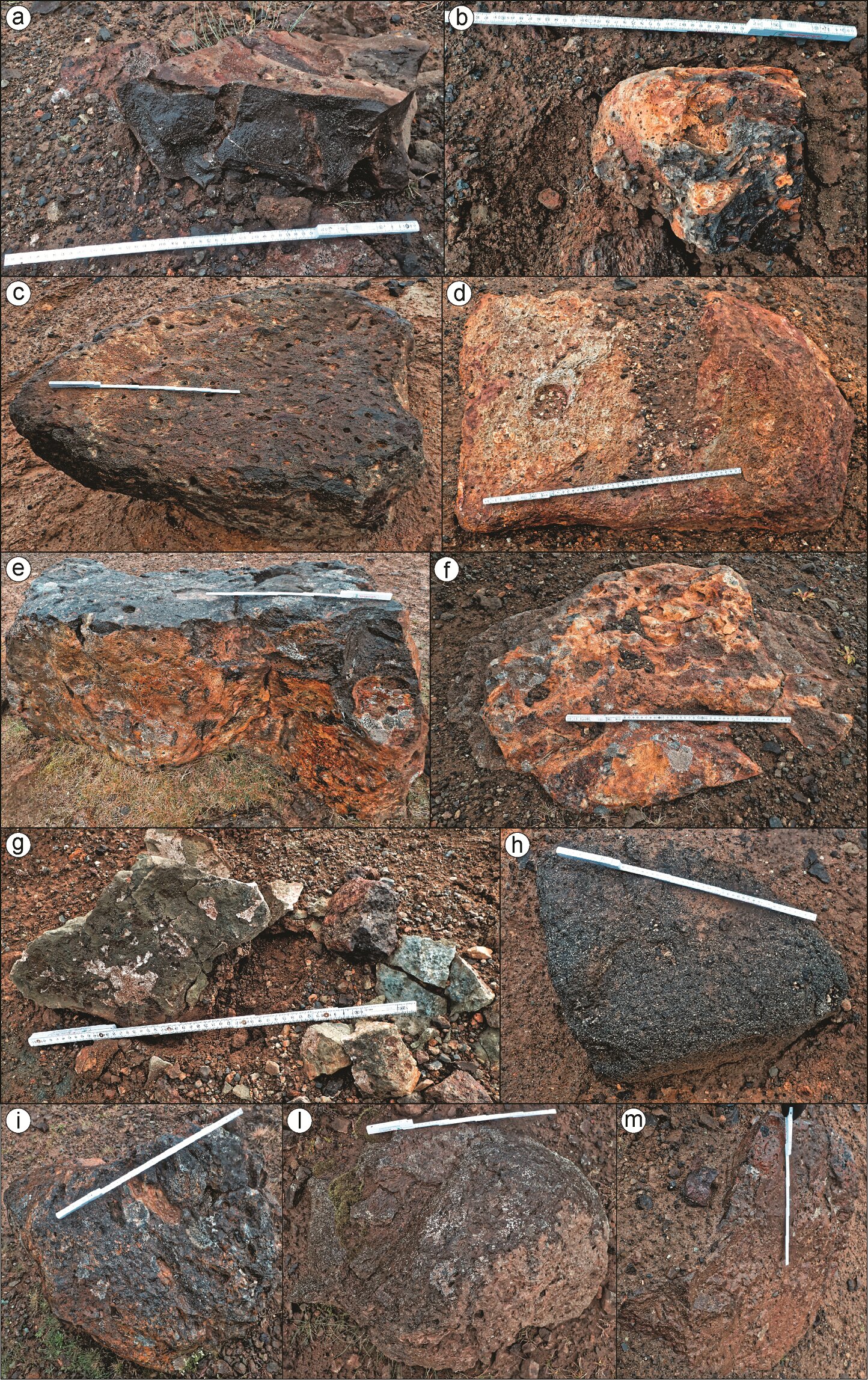


**Fig. ESM 3c:** Main lithologies of the coarse ballistic blocks (>25 cm) from the south-eastern sector: **a‒f, i, m** variably altered non-vesicular to vesicular lava; to be noticed the alteration mineral filling the rock pores; **g** altered green lavas and brecciated clasts; **h** highly altered tuff; **l** hyaloclastite tuff. The ruler used as scale in the pictures is 50 cm long.


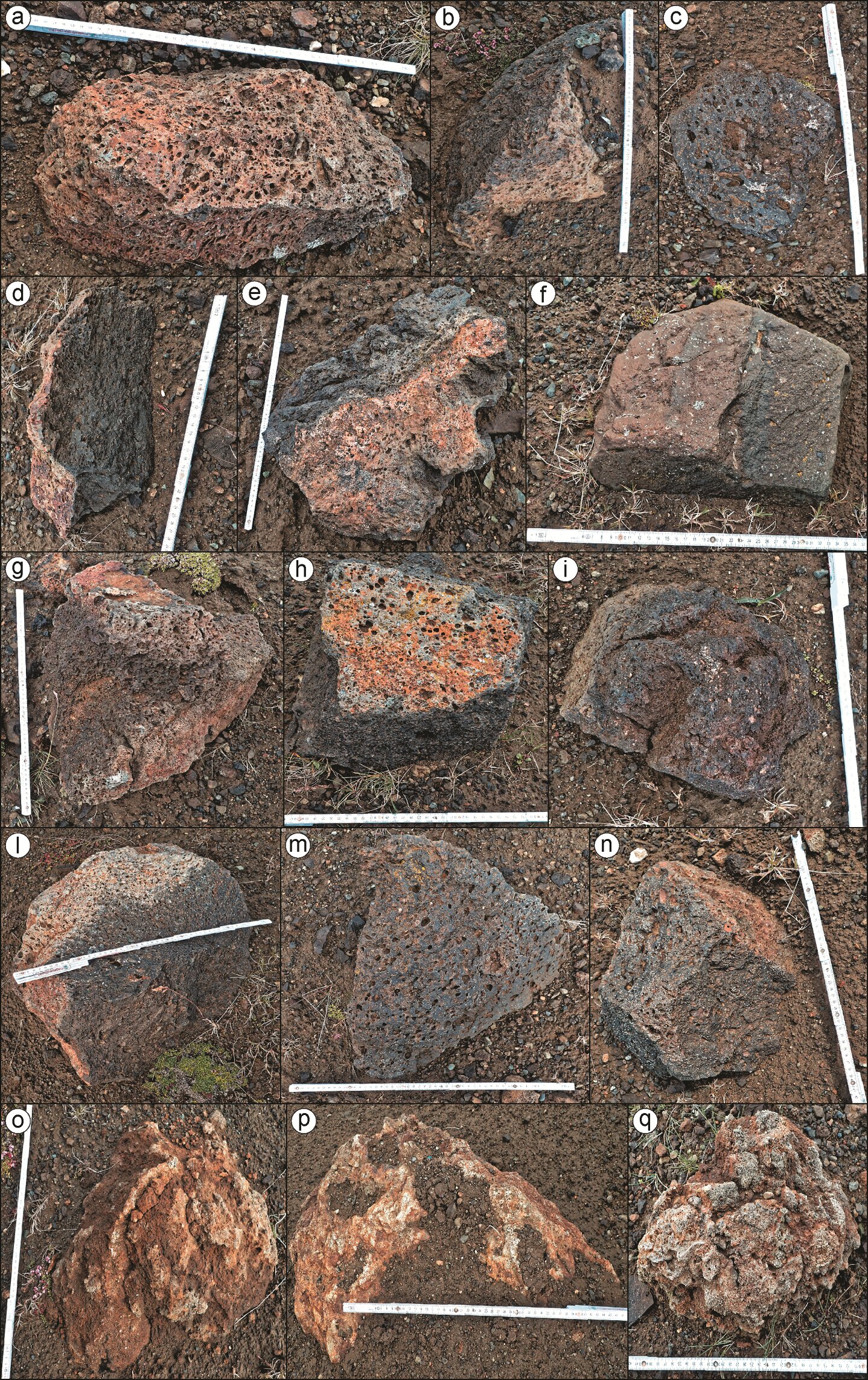


**Fig. ESM 3d:** Main lithologies of the coarse ballistic blocks (>25 cm) from the southern sector: **a‒q** variably altered non-vesicular to vesicular lava; to be noticed that samples a‒q are highly fractured and altered. The ruler used as scale in the pictures is 50 cm long.


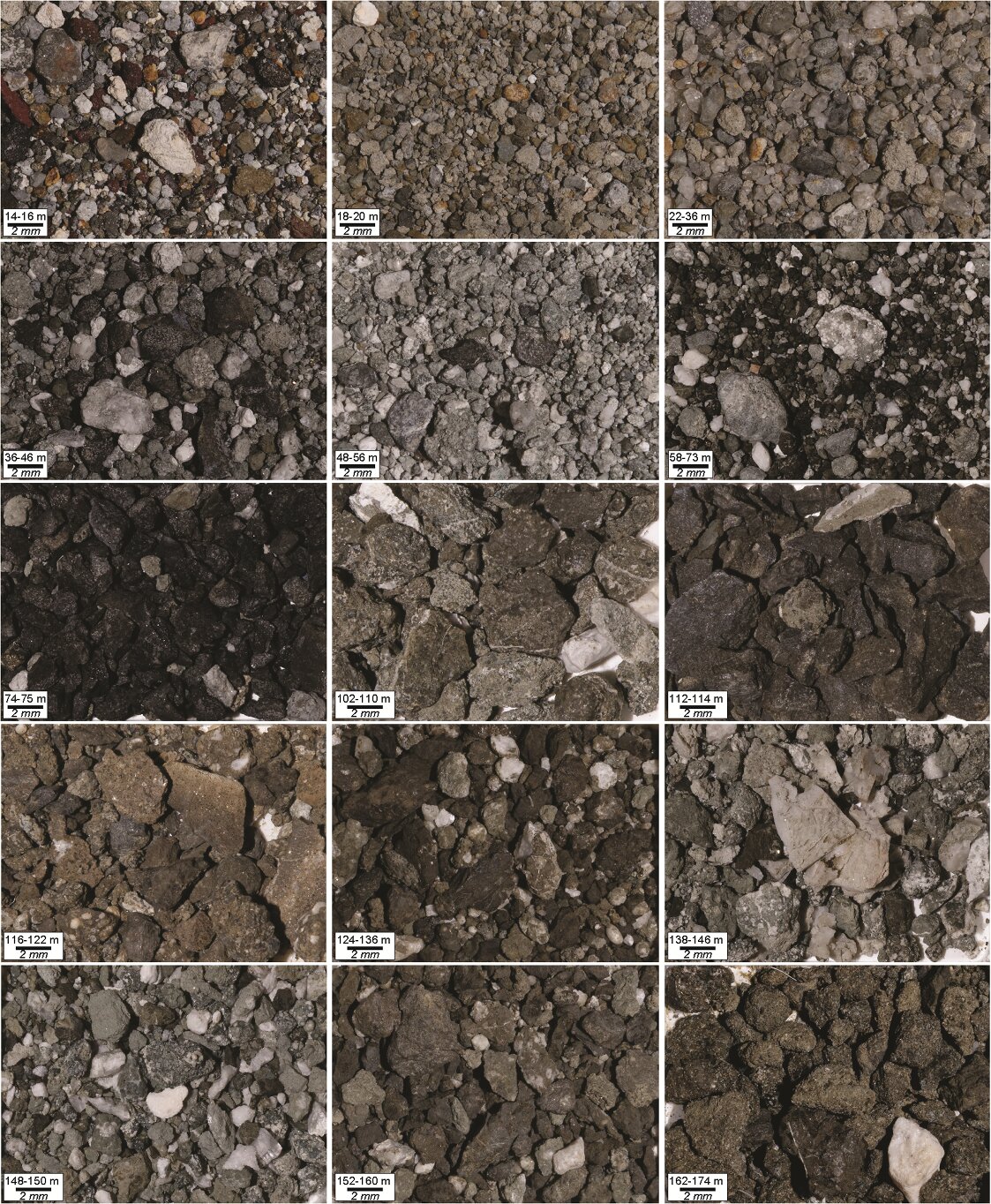


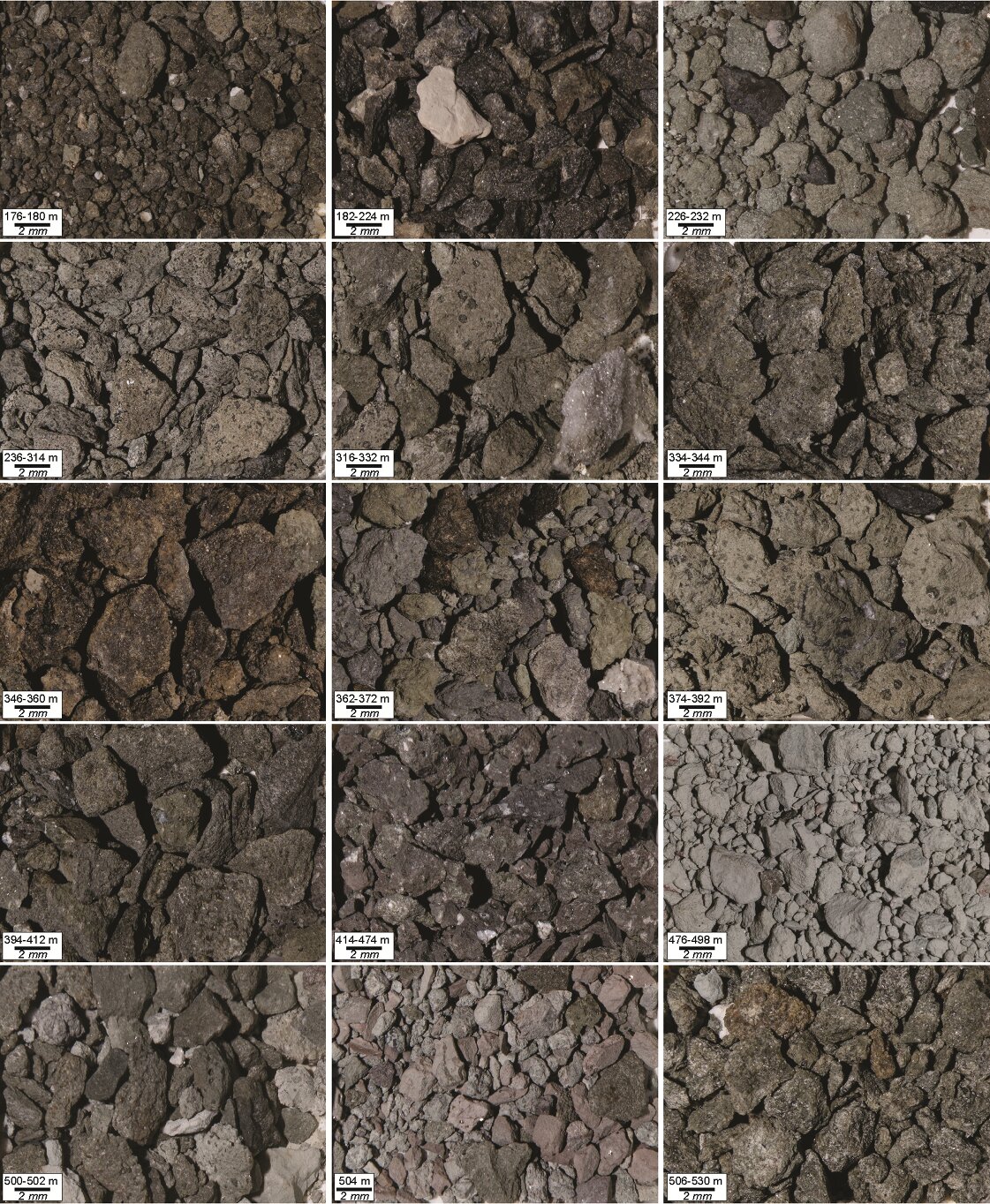


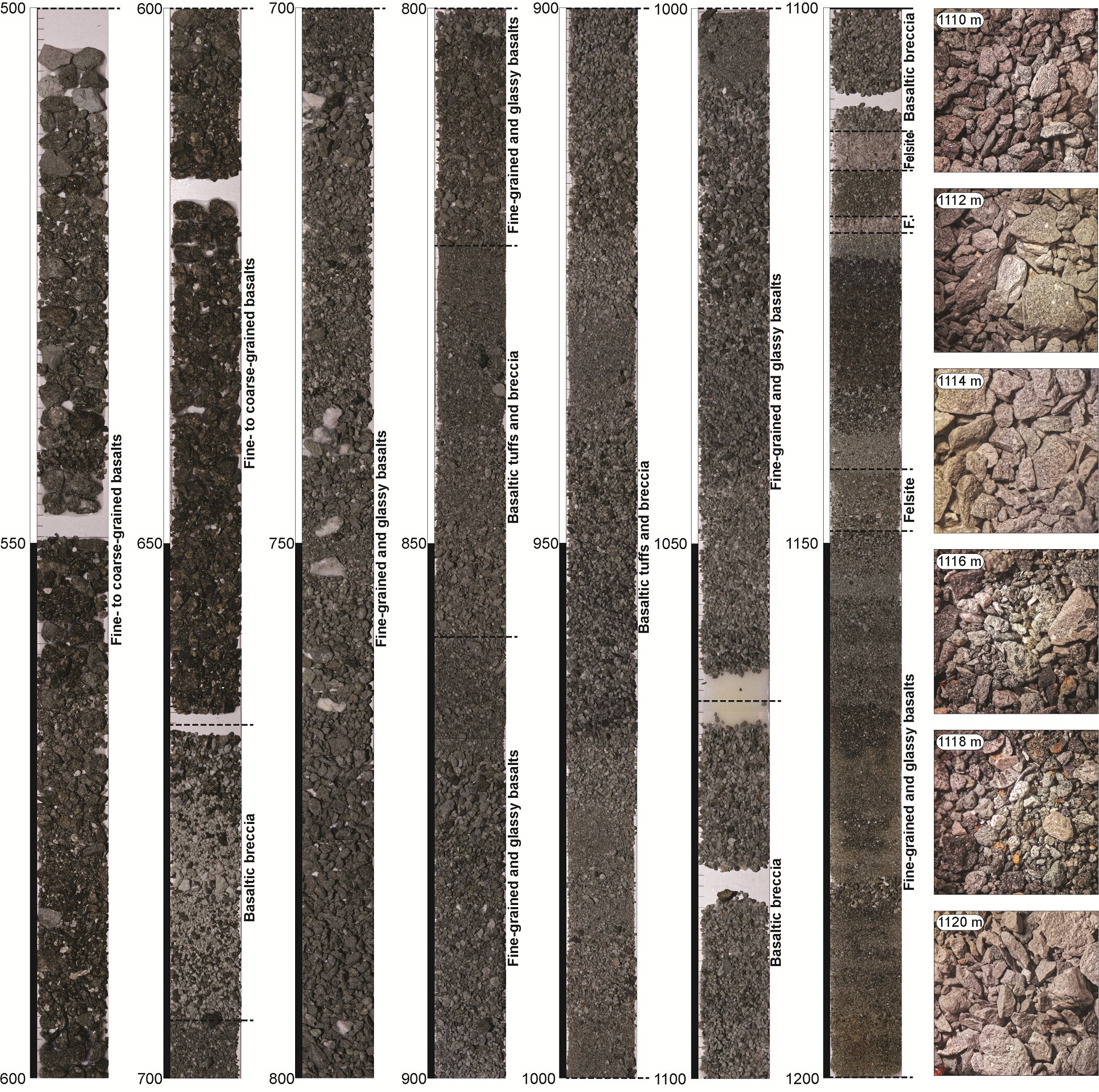


**Fig. ESM 4:** Representative lithologies from the main geological units drilled at K-36: **a** *14–100 m depth*: Oxidized scoria levels in the shallow (<20 m) ground and basaltic tuffs overlie a series of Holocene lavas intercalated with hyaloclastite consisting of mixed aphanitic basalt breccias and tuff; **a-b** *100–234 m depth*: Consolidated and greenish altered basaltic hyaloclastite (fine and medium grained tuffs) intercalated with lavas and minor breccias; **b-c** *234–822 m depth*: Thick sequence dominated by moderately altered basaltic lavas; predominantly medium-coarse grained basalt but also fine-medium grained basalt with intercalated aphanitic basalt and basaltic breccia. Between 470 and 490 m is light green moderately altered hyaloclastite, consisting of fine- to medium grained, aphyric, basaltic tuff; *822–1008 m depth*: Light grey to light green highly altered hyaloclastite, mostly made of fine-to-medium grained, aphyric basaltic tuff; *1008–1152 m depth*: Basaltic and basalt andesitic lava sequence. White felsite fragments were recovered in the drilling interval 1112‒1122 m and 1146–1152 m depth, with no clear evidence of intrusive contact with the host basalt. *Below 1152 m*: dyke intrusive rocks can be found.

**
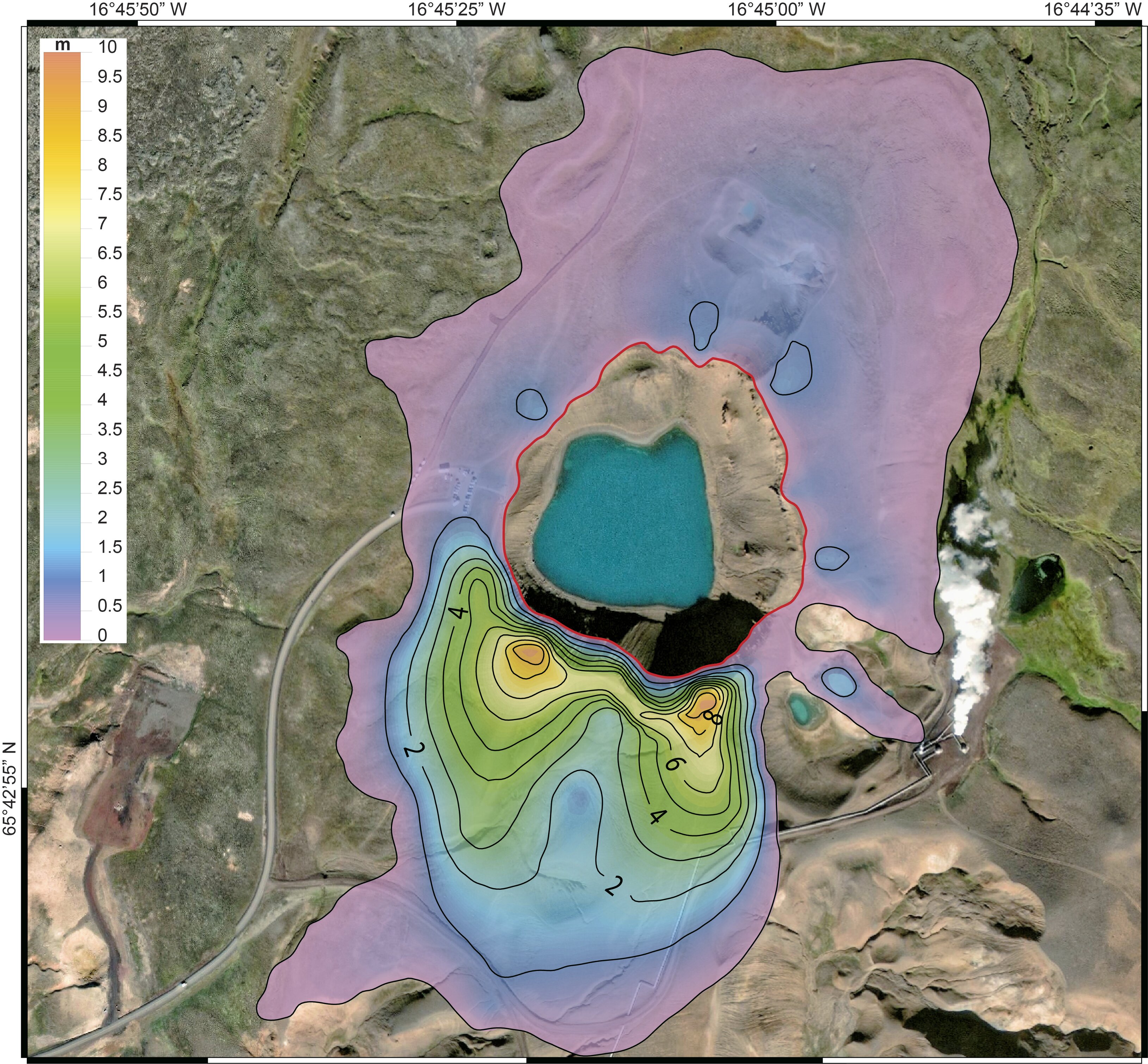
**

**Fig. ESM 5:** Satellite image (Google Earth™, 2016) of Viti crater and surrounding areas, with overimposed digital model and isopach showing the distribution and volume of the Viti Breccia 5. The isopach have been extrapolated from the measured breccia thickens (breccia logs and sampling points shown in Fig. 2). The red line represent the approximate base of the scoria cone deposits.
